# The accumulation of progerin underlies the loss of aortic smooth muscle cells in Hutchinson-Gilford progeria syndrome

**DOI:** 10.1038/s41419-025-07853-0

**Published:** 2025-07-24

**Authors:** Paul H. Kim, Joonyoung R. Kim, Patrick J. Heizer, Hyesoo Jung, Yiping Tu, Ashley Presnell, Julia Scheithauer, Rachel G. Yu, Stephen G. Young, Loren G. Fong

**Affiliations:** 1 Department of Medicine, University of California, Los Angeles, Los Angeles, CA USA; 2 Department of Human Genetics, University of California, Los Angeles, Los Angeles, CA USA

**Keywords:** Aortic diseases, Phosphorylation

## Abstract

Hutchinson-Gilford progeria syndrome (HGPS) is caused by progerin, an internally truncated prelamin A that does not undergo the ZMPSTE24 processing step that releases prelamin A’s farnesylated carboxyl terminus; consequently, progerin remains farnesylated. Progerin and full-length farnesyl-prelamin A are equivalent in their abilities to disrupt the nuclear lamina and trigger nuclear membrane ruptures and cell death, but they differ markedly in their abilities to cause arterial pathology. In HGPS mice (*Lmna*^G609G^), progerin causes loss of aortic smooth muscle cells (SMCs) by 12 weeks, whereas farnesyl-prelamin A in *Zmpste24*^−/−^ mice does not trigger SMC loss—even at 21 weeks. In young mice, farnesyl-prelamin A levels in *Zmpste24*^−/−^ aortas and progerin levels in *Lmna*^G609G^ aortas are identical; however, progerin levels in *Lmna*^G609G^ aortas increase progressively with age, whereas farnesyl-prelamin A levels in *Zmpste24*^−/−^ aortas remain the same or decline. SMC loss in *Zmpste24*^−/−^ aortas occurs only with supraphysiologic levels of prelamin A synthesis (mimicking the accumulation of progerin). AKT activity (which mediates prelamin A phosphorylation and triggers prelamin A turnover) is lower in *Lmna*^G609G^ aortas than in wild-type or *Zmpste24*^−/−^ aortas. Our studies show that the progressive accumulation of progerin in the aorta underlies the arterial pathology in HGPS.

## Introduction

Hutchinson-Gilford progeria syndrome (HGPS) is a pediatric progeroid disorder caused by point mutations that optimize a cryptic splice-donor site in exon 11 of *LMNA* (the gene for prelamin A and lamin C), resulting in aberrant mRNA splicing and the production of a prelamin A transcript lacking the last 150 nucleotides of exon 11 [1, 2]. That transcript results in the production of a mutant prelamin A, commonly called progerin, containing an internal deletion of 50 amino acids. Children with HGPS appear normal at birth, but they soon develop disease phenotypes that resemble physiologic aging [3–5]. Children with HGPS typically die during their teenage years from heart attacks or strokes (due to occlusive lesions in the cerebral and coronary arteries).

The products of the *LMNA* gene, prelamin A (the precursor to mature lamin A) and lamin C, are produced by alternative splicing [6]. The lamin C transcript contains exon 1–10 sequences; prelamin A contains exon 1–12 sequences. Lamin C is identical to prelamin A through the first 566 amino acids but contains six unique amino acids at its C terminus. Prelamin A contains 98 unique C-terminal amino acids and terminates with a *CaaX* motif, which triggers protein farnesylation, followed by three additional processing steps [endoproteolytic release of the last three residues by RCE1, methylation by ICMT, and cleavage of the last 15 amino acids by ZMPSTE24 [7]]. The ZMPSTE24-mediated processing step results in the production of mature lamin A.

The internal 50-amino acid deletion in progerin leaves its *CaaX* motif intact. Therefore, it does not affect protein farnesylation, C-terminal clipping, or methylation, but it eliminates the sequences required for ZMPSTE24 cleavage [8]. Thus, progerin is farnesylated and methylated but cannot be processed to mature lamin A. Progerin’s C-terminal farnesylcysteine methyl ester enhances interactions with the inner nuclear membrane and limits the mobility of progerin. Fluorescence recovery studies following photobleaching revealed that the mobility of progerin is similar to that of lamin B1 (which is farnesylated and methylated) but slower than mature lamin A (which lacks the farnesyl lipid anchor) [9].

Progerin is the culprit molecule in disease pathogenesis. When progerin is expressed in cultured cells, it triggers misshapen nuclei, DNA damage, and cell senescence; these same abnormalities are observed in fibroblasts from patients with HGPS [1, 9–12]. When progerin is expressed in mice, it triggers multiple disease phenotypes, including alopecia, failure-to-thrive, bone fractures, loss of adipose tissue, and progressive weight loss [13–15]. In addition, it causes the loss of medial smooth muscle cells (SMCs) in specific regions of the aorta such as the inner curvature of the ascending thoracic aorta and aortic arch, and arterial branches of the aortic arch [16, 17]. Loss of medial SMCs has also been documented in autopsy studies of aortas from patients with HGPS [18, 19]. Studies in mouse models of HGPS have provided valuable insights into the mechanisms of the vascular disease [15, 20–26]. Of note, the high progerin*–*low lamin B1 nuclear lamin profile in aortic SMCs (which weakens the functional integrity of the nuclear envelope), combined with the rhythmic stretching/relaxation occurring in the wall of the aorta [27], play important roles in SMC loss in the aorta.

Progerin’s ability to trigger disease phenotypes is often attributed to the fact that progerin, unlike mature lamin A or lamin C, contains a C-terminal farnesyl lipid anchor. In support of that view, a protein farnesyltransferase inhibitor (FTI) improves nuclear shape in progerin-expressing cells and ameliorates disease in knock-in and transgenic models of HGPS [11–13, 16, 28–30]. Also, disease phenotypes are absent in “knock-in” mice expressing a nonfarnesylated version of progerin [31]. Consistent with those observations, progerin expression in cultured cells results in the formation of a morphologically abnormal nuclear lamin meshwork [32, 33], whereas the meshwork formed by nonfarnesylated progerin is morphologically normal [33]. The importance of progerin’s internal deletion to its biological properties (other than eliminating ZMPSTE24-dependent processing) has attracted less attention.

Studies of *Zmpste24*^−/−^ mice have provided intriguing insights into the pathogenesis of progeria [34]. ZMPSTE24 deficiency abolishes the conversion of farnesyl-prelamin A to mature lamin A; consequently, tissues of *Zmpste24*^−/−^ mice contain lamin C and farnesyl-prelamin A but lack mature lamin A. In humans, ZMPSTE24 deficiency causes restrictive dermopathy, a neonatal-lethal progeroid syndrome characterized by markedly impaired growth, rigid skin, joint contractures, and bone fractures [35]. In mice, ZMPSTE24 deficiency causes many of the same phenotypes that have been documented in gene-targeted models of HGPS (e.g., alopecia, failure-to-thrive, bone fractures, loss of adipose tissue, and progressive weight loss); those phenotypes are severe and invariably result in premature death [36, 37]. However, in contrast to the gene-targeted models of HGPS mice, *Zmpste24*^−/−^ mice and *Lmna*^L648R/L648R^ knock-in mice (which eliminates ZMPSTE24-mediated prelamin A processing) do not exhibit loss of medial SMCs in the aorta [15, 38].

Why the HGPS mouse models, but not *Zmpste24*^−/−^ mice, exhibit vascular pathology is unknown, but this difference implies that progerin and full-length farnesyl-prelamin A—despite both terminating with a farnesylcysteine methyl ester—could have very distinct effects in the aorta. In the current study, we investigated that possibility. We discovered that progerin accumulates with age in the aorta, whereas farnesyl-prelamin A levels remain the same or decline. This difference underlies their distinct capacities to cause arterial disease.

## Results

### Loss of vascular SMCs in the proximal ascending thoracic aorta occurs in HGPS knock-in mice but not *Zmpste24*^−/−^ mice

**Fig. 1:**
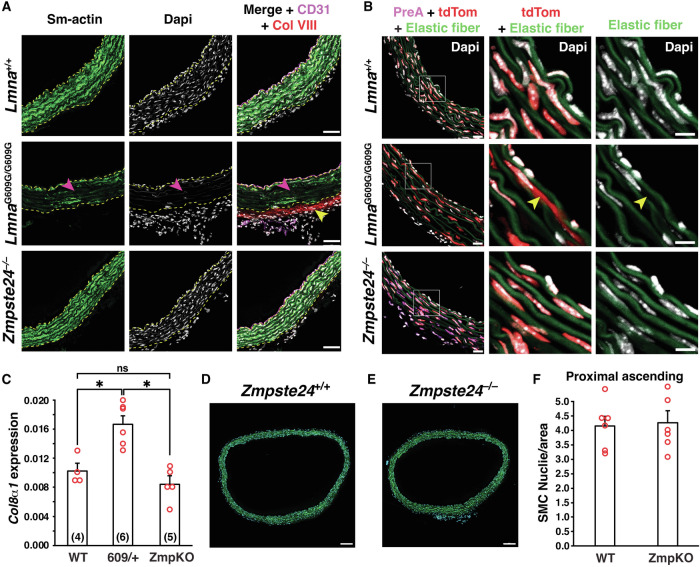
Loss of aortic smooth muscle cells (SMCs) occurs in HGPS knock-in mice but not *Zmpste24*^–/–^ mice. **A** Confocal fluorescence microscopy images of the proximal ascending aorta from 16-week-old *Lmna*^+/+^, *Lmna*^G609G/G609G^, and *Zmpste24*^−/−^ mice stained with antibodies against smooth muscle actin (Sm-actin, *green*), collagen type VIII (Col VIII, *red*), and CD31 (*magenta*). Nuclei were stained with Dapi (*white*). Scale bar, 50 µm. *Yellow* dotted lines mark the borders of the medial layer. *Red* arrowheads point to areas with reduced Sm-actin staining and reduced numbers of SMC nuclei. The *yellow* arrowhead points to collagen type VIII staining in the adventitia. Images of the entire tissue sections are shown in Fig. S1A. **B** Confocal fluorescence microscopy images of the proximal ascending aorta from 13-week-old *Lmna*^+/+^ and *Lmna*^G609G/G609G^ mice and a 21-week-old *Zmpste24*^−/−^ mouse, all expressing a nuclear-targeted tdTomato transgene, stained with an antibody against farnesyl-prelamin A. The boxed regions are shown at higher magnification in the middle and far-right columns. The images show Dapi (*white*), elastic fibers (*green*), tdTomato (tdTom; *red*), and farnesyl-prelamin A (PreA; *magenta*). The *yellow* arrowhead (middle column) points to tdTomato outside of an SMC nucleus. Scale bar, 10 µm. Images of the entire tissue sections are shown in Fig S1B. **C** Expression of *Col8α1* in aortas from 21-week old *Lmna*^+/+^, *Lmna*^G609G/+^, and *Zmpste24*^−/−^ mice. Mean ± SEM. The numbers of mice are shown in parentheses. ANOVA. *P < 0.01. Representative microscopy images of sections from the ascending thoracic aorta [stained with Dapi (*blue*)] from a 21-week-old *Zmpste24*^+/+^ mouse (**D**) and a *Zmpste24*^−/−^ mouse (**E**) Scale bar, 100 µm. **F** Bar graph showing the numbers of nuclei (relative to area) in the proximal ascending thoracic aorta in 21-week-old *Zmpste24*^+/+^ (WT) and *Zmpste24*^−/−^ (ZmpKO) mice. Mean ± SEM (*n* = 5 mice/group). Student’s *t* test. *P* = 0.84. Images of the entire tissue sections used for quantification are shown in Fig. S2.

We examined aortas from wild-type mice (*Lmna*^+/+^), *Zmpste24*^−/−^ mice, and a gene-targeted knock-in mouse model of HGPS (*Lmna*^G609G/G609G^). Since the loss of vascular SMCs in mouse models of HGPS is not widespread but occurs mainly in specific regions (*i.e*., the inner curvature of the proximal ascending aorta and aortic arch, and at the major arterial branches of the aortic arch) [15], we examined SMC loss at the proximal ascending thoracic aorta. The HGPS vascular disease is most severe at this location and is highly reproducible [15]. Cross sections were stained with antibodies against α-smooth muscle actin [a marker of SMCs], CD31 (an endothelial cell marker), and collagen type VIII [a marker of HGPS-associated adventitial fibrous; [15]]. Confocal microscopy images of the inner curvature of the ascending aorta in *Lmna*^G6096G/G609G^ mice revealed reduced α-smooth muscle actin staining, reduced numbers of SMCs, and increased collagen type VIII staining of the adventitia (Figs. 1A and S1A). In contrast, aortas of *Zmpste24*^−/−^ mice were normal, indistinguishable from those in *Lmna*^+/+^ mice. Consistent with those findings, nuclear membrane ruptures (identified by escape of a nuclear-targeted tdTomato into the cytoplasm) were frequent in the medial SMCs of *Lmna*^G6096G/G609G^ aortas but absent in *Zmpste24*^−/−^ aortas (Figs. 1B and S1B). As expected, farnesyl-prelamin A was detected in aortas of *Zmpste24*^−/−^ mice but not in *Lmna*^+/+^ or *Lmna*^G6096G/G609G^ mice (Figs. 1B and S1B). *Col8α1* gene expression, a marker of mouse HGPS vascular disease [15], was increased in *Lmna*^G609G/+^ mice but not in *Zmpste24*^−/−^ mice (Fig. 1C). The numbers of medial SMCs in the aorta were the same in *Zmpste24*^−/−^ mice and in wild-type mice (Figs. 1D–F and S2).

### Progerin and farnesyl-prelamin A have similar effects in cultured SMCs

**Fig. 2:**
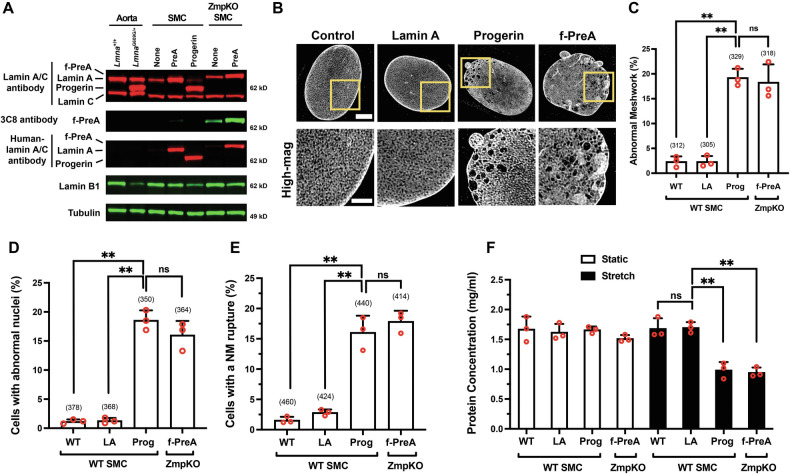
Progerin and farnesyl-prelamin A have similar effects in cultured SMCs. **A** Western blot comparing the expression of nuclear lamins in the mouse aorta and in mouse SMCs expressing human lamin A, human progerin, and human farnesyl-prelamin A. Nuclear lamins were detected with antibodies that bind to mouse and human lamin A/C, farnesyl-prelamin A (clone 3C8), human lamin A/C, and lamin B1. Tubulin was measured as a loading control. **B** Representative high-resolution confocal microscopy images showing the protein meshworks formed by human versions of lamin A, progerin, and farnesyl-prelamin A (f-PreA). Scale bar, 5 µm. The boxed regions are shown at higher magnification below. Scale bar, 2 µm. **C** Nuclei with an abnormal meshwork in SMCs expressing human lamin A (LA), progerin (Prog), and farnesyl-prelamin A (f-PreA). Mean ± SEM (*n* = 3 experiments). ANOVA. **P < 0.01. ns, not significant. **D** Abnormal nuclear shape in SMCs expressing human versions of lamin A, progerin, and farnesyl-prelamin A. Mean ± SEM (*n* = 3 experiments). ANOVA. **P < 0.01. ns, not significant. **E** Nuclear membrane (NM) ruptures in SMCs expressing human versions of lamin A, progerin, and farnesyl-prelamin A. Mean ± SEM (*n* = 3 experiments). ANOVA. **P < 0.01. ns, not significant. **F** Cell death in SMCs expressing human versions of lamin A, progerin, or farnesyl-prelamin A. SMCs were cultured on PDMS membranes and exposed to static (open bars) or cyclical stretching conditions (closed bars) for 24 h. The fraction of cells remaining on the membranes were quantified by measuring protein concentration. Mean ± SEM (*n* = 3 experiments). ANOVA. **P < 0.01. For the data reported in (**C**–**E**), the number of nuclei or cells examined are shown in parentheses.

We used doxycycline (Dox)-inducible constructs to create mouse SMC clones that expressed human versions of progerin (Prog-SMC), mature lamin A (PreA-SMC), and farnesyl-prelamin A (PreA-ZMPKO-SMCs). The levels of nuclear lamin expression in the SMC clones were adjusted to match the amounts of progerin in aortas of *Lmna*^G609G/+^ mice (Fig. 2A). The expression of farnesyl-prelamin A in PreA-ZMPKO-SMCs was confirmed by western blotting with monoclonal antibody (mAb) 3C8, which binds preferentially to the farnesylated form of prelamin A [39] (Fig. 2A).

We next examined whether progerin and farnesyl-prelamin A have similar biologic effects. First, we examined the structures of the progerin and farnesyl-prelamin A nuclear lamin meshworks by high-resolution microscopy. Previous studies showed that progerin forms an abnormal meshwork with large gaps and that progerin has a dominant-negative effect on the lamin B1 meshwork [33]. The nuclear lamin meshworks were examined after staining with a human lamin A/C–specific antibody that binds to all A-type nuclear lamins [33]. The mature lamin A in PreA-SMCs formed a meshwork with small, uniform gaps and was morphologically indistinguishable from the mouse lamin A/C meshwork in wild-type SMCs (Fig. 2B). In contrast, progerin and farnesyl-prelamin A both formed abnormal meshworks (with large and irregular-sized gaps) in SMCs (~17%) (Fig. 2B, C). Aside from triggering similar morphological abnormalities in the nuclear lamin meshwork, progerin and farnesyl-prelamin A triggered very similar frequencies of misshapen nuclei and nuclear membrane (NM) ruptures (Fig. 2D). Misshapen nuclei were present in 18% of Prog-SMCs and in 16% of PreA-ZMPKO-SMCs but were present in less than 1% of PreA-SMCs (≥350 cells/group). Similarly, NM ruptures [detected by live-cell imaging [21]] were present in 16% of Prog-SMCs and 18% in PreA-ZMPKO-SMCs, but were present in less than 3% of PreA-SMCs (≥414 cells/group) (Fig. 2E). Finally, we compared the effects of progerin and farnesyl-prelamin A on SMC survival after stretching (to mimic the cyclical stretch in the aorta). Earlier studies showed that progerin causes cell death when SMCs are subjected to cyclical stretching [15]. The dead cells detach from the stretched membranes, as judged by trypan blue staining. To quantify the amount of live cells remaining on the membrane, we measured cell protein. Progerin and farnesyl-prelamin A increased the frequency of cell death to a similar degree (Fig. 2F). Cell death was observed in 40% of Prog-SMCs and 38% of PreA-ZMPKO-SMCs but in less than 1% of PreA-SMCs.

### Farnesyl-prelamin A does not accumulate with age in the thoracic aorta of *Zmpste24*^−/−^ mice

**Fig. 3:**
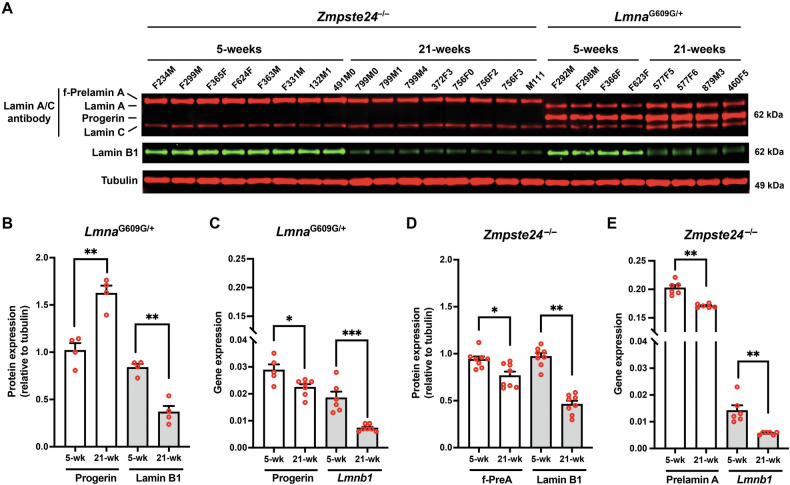
Farnesyl-prelamin A does not accumulate with age in the thoracic aorta of *Zmpste24*^–/–^ mice. **A** Western blot comparing the expression of farnesyl-prelamin A, progerin, and lamin B1 in aortas from young and old *Zmpste24*^−/−^ and *Lmna*^G609G/+^ mice. Tubulin was measured as a loading control. The ages and mouse IDs are shown above each sample. **B** Bar graph showing progerin and lamin B1 protein expression (relative to tubulin) in aortas from young and old *Lmna*^G609G/+^ mice. Mean ± SEM (*n* = 4 mice/group). Student’s *t* test. **P < 0.01. **C** Quantitative RT-PCR studies showing progerin and *Lmnb1* transcript levels in aortas from young and old *Lmna*^G609G/+^ mice. Mean ± SEM (*n* = 5–7 mice/group). Student’s *t* test. *P < 0.05. ***P < 0.001. **D** Bar graph showing farnesyl-prelamin A (f-PreA) and lamin B1 expression (relative to tubulin) in aortas from young and old *Zmpste24*^−/−^ mice. Mean ± SEM (*n* = 8 mice/group). Student’s *t* test. *P < 0.05. **P < 0.01. **E** Quantitative RT-PCR studies showing prelamin A and *Lmnb1* transcript levels in aortas from young and old *Zmpste24*^−/−^ mice. Mean ± SEM (*n* = 6 mice). Student’s *t* test, **P < 0.01.

We hypothesized that the striking differences in aortic pathology in *Lmna*^G609G/G609G^ and *Zmpste24*^−/−^ mice (despite identical toxicities of progerin and farnesyl-prelamin A in cultured SMCs) could be due to different amounts of progerin and farnesyl-prelamin A in aortas. To explore that possibility, we examined aortas of 5- and 21-week-old *Zmpste24*^−/−^ and *Lmna*^G609G/+^ mice (Fig. 3A). For these time course studies, separate cohorts of mice were used at the different time points. Consistent with earlier findings [21], the levels of progerin in the aortas of *Lmna*^G609G/+^ mice increased with age, whereas the levels of lamin B1 declined. Levels of progerin increased by 62% between 5 and 21 weeks of age, and lamin B1 levels fell by 57% (*n* = 4 mice/group) (Fig. 3B). The age-related increase in progerin levels could not be explained by higher transcript levels; progerin transcript levels in the aorta fell by 22% with age (Fig. 3C). *Lmnb1* transcripts in *Lmna*^G609G/+^ mice declined by 61% (Fig. 3C). In parallel, we examined aortas in age-matched *Zmpste24*^−/−^ mice (*n* = 8 mice/group). In young animals, the levels of farnesyl-prelamin A in *Zmpste24*^−/−^ mice were equivalent to the levels of progerin in *Lmna*^G609G/+^ mice. In contrast to the age-related increase in progerin levels in *Lmna*^G609G/+^ aortas, the levels of farnesyl-prelamin A in *Zmpste24*^−/−^ aortas decreased by 19% (Fig. 3D). Lamin B1 protein levels in *Zmpste24*^−/−^ aortas decreased by 53% (Fig. 3D). Prelamin A transcript levels in *Zmpste24*^−/−^ aortas fell by 15% at 21 weeks; *Lmnb1* transcript levels were reduced by 61% (Fig. 3E). The levels of lamin C protein also changed with age. Lamin C levels increased 76% in *Lmna*^G609G/+^ mice (*n* = 4; *P* < 0.02) while lamin C levels decreased by 22% in *Zmpste24*^−/−^ mice (*n* = 8; *P* < 0.01).

### Progerin causes accumulation of A-type nuclear lamins in the thoracic aorta and heart

**Fig. 4:**
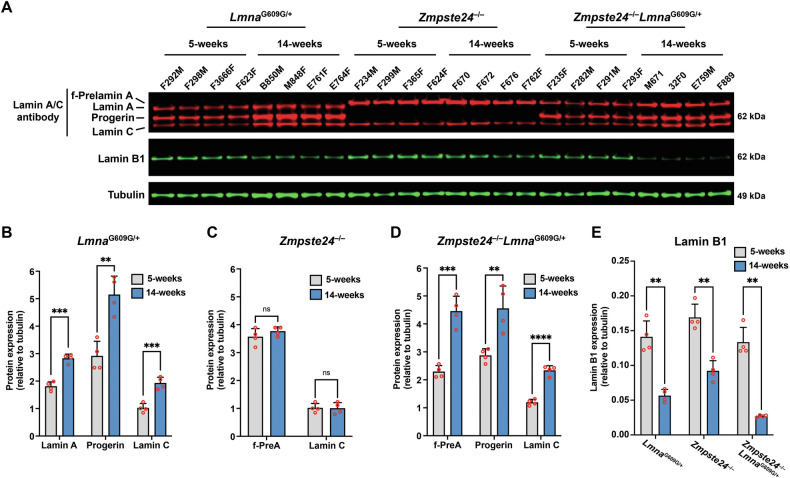
Progerin causes the accumulation of the A-type nuclear lamins in the thoracic aorta. **A** Western blot comparing the expression of lamin A, lamin C, farnesyl-prelamin A, progerin, and lamin B1 in aortas from 5- and 14-week-old *Lmna*^G609G/+^, *Zmpste24*^−/−^, and *Zmpste24*^−/−^*Lmna*^G609G/+^ mice. Tubulin was measured as a loading control. The ages and mouse IDs are shown above each sample. **B** Bar graph showing lamin A, progerin, and lamin C expression (relative to tubulin) in aortas from young and old *Lmna*^G609G/+^ mice. Mean ± SEM (*n* = 4 mice/group). Student’s *t* test. **P < 0.01. ***P < 0.001. **C** Bar graph showing farnesyl-prelamin A (f-PreA) and lamin C expression (relative to tubulin) in aortas from young and old *Zmpste24*^−/−^ mice. Mean ± SEM (*n* = 4 mice/group). Student’s *t* test. ns, not significant. **D** Bar graph showing f-PreA, progerin, and lamin C expression (relative to tubulin) in aortas from young and old *Zmpste24*^−/−^*Lmna*^G609G/+^ mice. Mean ± SEM (*n* = 4 mice/group). Student’s *t* test. **P < 0.01. ***P < 0.001. ****P < 0.0001. **E** Bar graph showing lamin B1 expression (relative to tubulin) in aortas from young and old *Lmna*^G609G/+^, *Zmpste24*^−/−^, and *Zmpste24*^−/−^*Lmna*^G609G/+^ mice. Mean ± SEM (*n* = 4 mice/group). Student’s *t* test. **P < 0.01.

The fact that progerin levels increased with age in *Lmna*^G609G/+^ aortas without a corresponding increase in progerin transcripts [[21] and Fig. 3B, C] raised the possibility that progerin itself turns over more slowly or progerin alters the turnover of nuclear lamins. To explore this, we isolated aortas from separate cohorts of young (5 week) and old (14 week) *Lmna*^G609G/+^ mice and quantified aortic levels of lamin A, lamin C, and progerin. The levels of all three A-type nuclear lamins increased with age (Fig. 4A, B). We also examined aortas in age-matched *Zmpste24*^−/−^ mice. The A-type nuclear lamins in *Zmpste24*^−/−^ mice (farnesyl-prelamin A and lamin C) did not increase with age (Fig. 4A, C). To test whether the expression of progerin was responsible for the age-related increase in A-type nuclear lamin levels in the aorta, we bred *Zmpste24*-deficient mice that expressed progerin (*Zmpste24*^−/−^*Lmna*^G609G/+^). In contrast to the findings in *Zmpste24*^−/−^ mice, the levels of farnesyl-prelamin A and lamin C in *Zmpste24*^−/−^*Lmna*^G609G/+^ aortas increased with age by 1.9- and 2-fold, respectively (Fig. 4A, D). Quantitative PCR studies revealed that prelamin A, progerin, and lamin C transcript levels in the aorta did not increase with age (Fig. S3B–D). Thus, the age-related increase in A-type nuclear lamins in *Lmna*^G609G/+^ and *Zmpste24*^−/−^*Lmna*^G609G/+^ aortas could not be explained by increased levels of *Lmna* transcripts. In these studies, aortas were examined at 14 weeks rather than 21 weeks of age because the disease phenotypes occurred earlier in *Zmpste24*^−/−^*Lmna*^G609G/+^ mice than in *Zmpste24*^−/−^ mice (Fig. S3A). The shorter time interval likely explains why we did not observe a decrease in the levels of farnesyl-prelamin A in older *Zmpste24*^−/−^ mice (see Fig. 3D). Lamin B1 protein levels in aortas of *Lmna*^G609G/+^, *Zmpste24*^−/−^, and *Zmpste24*^−/−^*Lmna*^G609G/+^ mice all decreased with age (by 60%, 46%, and 79%, respectively; *P* < 0.01) (Fig. 4E).

We also examined the impact of progerin on A-type nuclear lamins in the heart from the same animals. Similar to the aorta, the amounts of A-type nuclear lamins in hearts of *Lmna*^G609G/+^ and *Zmpste24*^−/−^*Lmna*^G609G/+^ mice increased with age, whereas the amounts of A-type lamins remained stable in *Zmpste24*^−/−^ mice (Fig. S4A–D). Lamin B1 levels in hearts of *Lmna*^G609G/+^ and *Zmpste24*^−/−^*Lmna*^G609G/+^ mice decreased with age or remained the same (Fig. S4E). Transcript levels for the A-type nuclear lamins in hearts of *Lmna*^G609G/+^ and *Zmpste24*^−/−^*Lmna*^G609G/+^ mice did not increase with age (Fig. S3E–G).

### Loss of SMCs in the proximal ascending thoracic aortas of *Zmpste24*-deficient mice can be triggered by raising the levels of prelamin A production

**Fig. 5:**
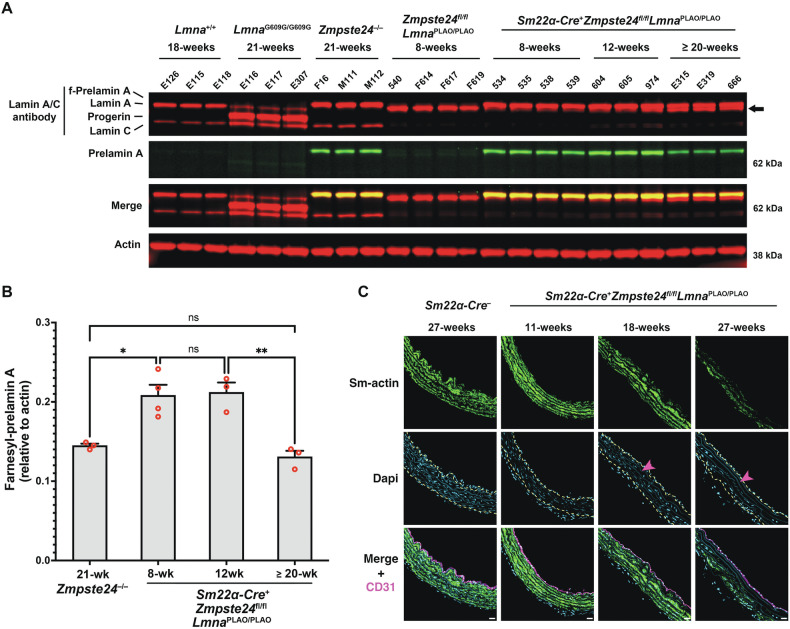
The loss of SMCs in the thoracic aortas of *Zmpste24*-deficient mice can be induced by increasing prelamin A production. **A** Western blot comparing the expression of lamin A, lamin C, farnesyl-prelamin A, progerin, and lamin B1 in aortas from wild-type, *Lmna*^G609G/G609G^, *Zmpste24*^−/−^, *Zmpste24*^fl/fl^*Lmna*^PLAO/PLAO^, and *Sm22α-CreZmpste24*^fl/fl^*Lmna*^PLAO/PLAO^ mice. Farnesyl-prelamin A was detected with monoclonal antibody 3C8. Actin was measured as a loading control. The ages and mouse IDs are shown above each sample. The arrow points to mature lamin A in older *Sm22α-CreZmpste24*^fl/fl^*Lmna*^PLAO/PLAO^ mice (detected with an anti-lamin A/C antibody). **B** Bar graph comparing the expression of farnesyl-prelamin A (detected with antibody 3C8) relative to actin in 21-week-old *Zmpste24*^−/−^ mice and *Sm22α-CreZmpste24*^fl/fl^*Lmna*^PLAO/PLAO^ mice at 8, 12, and ≥20 weeks of age. ANOVA. *P < 0.05. **P < 0.01. ns, not significant. **C** Confocal fluorescence microscopy images of the proximal ascending aorta from 11-, 18-, and 27-week-old *Sm22α-CreZmpste24*^fl/fl^*Lmna*^PLAO/PLAO^ mice stained with antibodies against smooth muscle actin (Sm-actin, *green*) and CD31 (*magenta*). As a control, images from a 27-week-old *Zmpste24*^fl/fl^*Lmna*^PLAO/PLAO^ mouse are shown. Nuclei were stained with Dapi (*blue*). Scale bar, 20 µm. *Yellow* dotted lines mark the borders of the medial layer. *Red* arrowhead points to an area with reduced Sm-actin staining and reduced numbers of SMC nuclei. Images of the entire sections are shown in Fig. S7. Images from additional 27-week-old *Sm22α-CreZmpste24*^fl/fl^*Lmna*^PLAO/PLAO^ mice are shown in Fig. S8.

We suspected that *Zmpste24*^−/−^ mice were protected from aortic SMC loss due to the absence of farnesyl-prelamin A accumulation with age. We reasoned that it might be possible to induce SMC loss in aortas of *Zmpste24*^−/−^ mice by increasing prelamin A production (so as to mimic the accumulation of progerin in *Lmna*^G609G/+^ mice). To test this, our initial plan was to breed *Zmpste24*^−/−^ mice harboring two “prelamin A–only” alleles (*Lmna*^PLAO/PLAO^), which channels the output of *Lmna* exclusively into prelamin A rather than into both lamin C and prelamin A [40]. In *Lmna*^PLAO/PLAO^ mice, the levels of lamin A are increased by ~40% (Fig. S5A, B). Unfortunately, however, this strategy was not useful in *Zmpste24*^−/−^ mice because the high levels of farnesyl-prelamin A expression in every tissue of *Zmpste24*^−/−^*Lmna*^PLAO/PLAO^ mice resulted in severe, multisystem disease and death by ~6–7 weeks of age. To circumvent that roadblock, we bred *Sm22α*-*CreZmpste24*^fl/fl^*Lmna*^PLAO/PLAO^ mice, which makes it possible to increase levels of farnesyl-prelamin A preferentially in SMCs. The expression of *Sm22α*-*Cre* in aortic SMCs was confirmed with a dual-fluorescence *Rosa*^nT-nG^ reporter allele (where tdTomato is expressed in the nucleus of cells but switches to EGFP after *Cre*-recombination) [41]. That approach makes it possible to visualize both *Cre*-positive and *Cre*-negative cells in the same sample. As expected, the two-color reporter revealed *Cre* expression in aortic SMCs of *Sm22α*-*Cre*-positive mice (Fig. S6A); however, not all vascular SMCs expressed the *Sm22α*-*Cre* transgene (*i.e*., tdTomato-positive SMCs persisted in the medial layer of the aorta). Nonetheless, farnesyl-prelamin A was detected in aortic SMCs by immunohistochemistry (Fig. S6B) and the levels were increased in *Sm22α*-*CreZmpste24*^fl/fl^*Lmna*^PLAO/PLAO^ mice. At 8 and 12 weeks of age, western blotting of separate cohorts of mice showed that the levels of farnesyl-prelamin A in the aorta were ~45% higher in *Sm22α*-*CreZmpste24*^fl/fl^*Lmna*^PLAO/PLAO^ mice than in *Zmpste24*^−/−^ mice. At 18 and 27 weeks of age, histopathology studies of *Sm22α*-*CreZmpste24*^fl/fl^*Lmna*^PLAO/PLAO^ mice revealed loss of aortic SMCs along with reduced smooth muscle actin staining (Figs. 5C, and S7, S8). Thus, increasing the levels of farnesyl-prelamin A in SMCs triggered aortic pathology. Interestingly, the aortic pathology developed even though the levels of farnesyl-prelamin A in 20-week-old *Sm22α*-*CreZmpste24*^fl/fl^*Lmna*^PLAO/PLAO^ mice were lower than the levels at 12 weeks of age (Fig. 5A, B). The decreased farnesyl-prelamin A levels were accompanied by increased amounts of mature lamin A. The latter findings likely reflect the incomplete expression of the *Sm22α*-*Cre* transgene in SMCs and survival of aortic SMCs that retained *Zmpste24* expression (see *Discussion*).

### Reduced phosphorylation of serine-404 in progerin

**Fig. 6:**
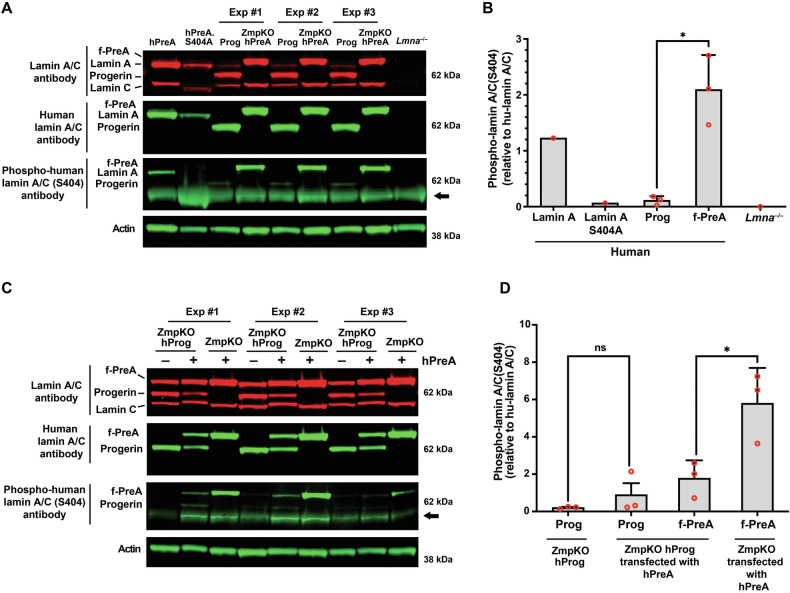
Reduced phosphorylation of serine-404 in progerin. **A** Western blot comparing the levels of serine-404 phosphorylation in human versions of lamin A, farnesyl-prelamin A (f-PreA), and progerin (Prog) expressed in mouse SMCs. Serine-404 phosphorylation was detected with an antibody against human lamin A/C phosphoserine-404. The specificity of the antibody was evaluated using extracts from SMCs expressing an S404A human lamin A mutant and *Lmna*^−/−^ SMCs. The levels of expression of the human nuclear lamin proteins were measured with an anti-human lamin A/C antibody. Actin was measured as a loading control. The arrow points to a nonspecific band produced with the human lamin A/C phosphoserine-404 antibody. **B** The bar graph compares the levels of serine-404 phosphorylation in the human versions of lamin A, lamin A-S404A, Prog, and f-PreA. Serine-404 phosphorylation was normalized to the level of human lamin protein expression. Mean ± SEM (*n* = 3). Student’s *t* test. *P < 0.05. **C** Western blot comparing the effects of progerin expression on the phosphorylation of serine-404 in human farnesyl-prelamin A. Human farnesyl-prelamin A synthesis was induced by transient transfection of human-prelamin A (hPreA) in *Zmpste24*^−/−^ SMCs or *Zmpste24*^−/−^ SMCs expressing progerin (ZmpKO hProg). Serine-404 phosphorylation in human farnesyl-prelamin A and progerin was detected as described in (**A**). The arrow points to a nonspecific band produced with the human lamin A/C phosphoserine-404 antibody. **D** The bar graph compares the effects of progerin expression on the levels of serine-404 phosphorylation in farnesyl-prelamin A. The levels of serine-404 phosphorylation were measured as described in (**B**). Mean ± SEM (*n* = 3). ANOVA. *P < 0.05. ns, not significant.

Earlier studies established that phosphorylation of prelamin A at serine-404 by AKT triggers prelamin A degradation by lysosomal enzymes [42]. Considering the differences in levels of progerin and farnesyl-prelamin A in older mice, we hypothesized that the age-related accumulation of progerin in *Lmna*^G609G/+^ mice might result from lower levels of serine-404 phosphorylation. To test this possibility, we compared the phosphorylation of serine-404 in human progerin and human farnesyl-prelamin A in SMCs by western blotting (with an antibody against human lamin A/C phosphoserine-404). The specificity of the antibody was confirmed in cells expressing a S404A-human lamin A mutant and in *Lmna*^−/−^ SMCs (Fig. 6A, B). The phosphoserine lamin A antibody bound avidly to farnesyl-prelamin A in PreA-ZMPKO-SMCs, whereas the binding of the antibody to progerin in Prog-SMCs was very low (~5% of the binding to farnesyl-prelamin A) (Fig. 6A, B). To determine if the reduced phosphorylation of progerin was due to progerin itself, we transiently expressed human prelamin A in ZMPKO-SMCs and in ZMPKO-SMCs that expressed progerin; we then assessed the phosphorylation of farnesyl-prelamin A at serine-404. In the ZMPKO-SMCs expressing progerin, the phosphorylation of farnesyl-prelamin A was significantly reduced (Fig. 6C, D). These studies suggest that progerin reduces AKT activity.

**Fig. 7:**
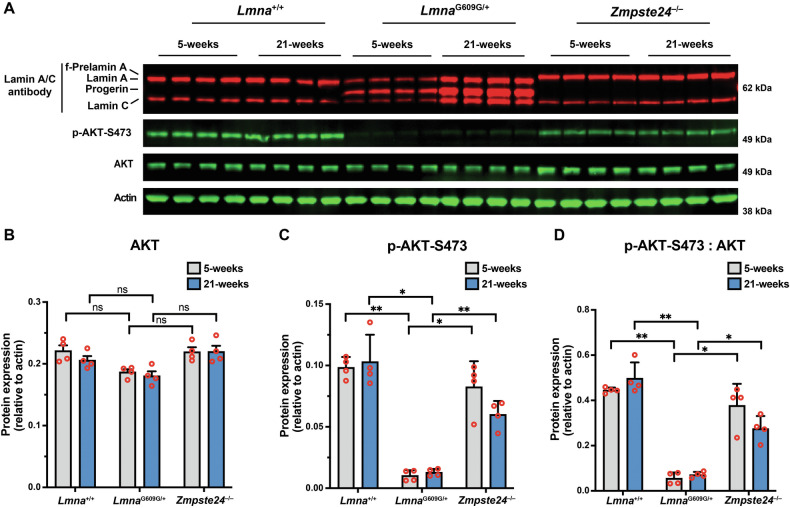
AKT activity is reduced in the thoracic aorta of *Lmna*^G609G/+^ mice. **A** Western blot comparing the levels of phosphorylated AKT at serine-473 (p-AKT-S473) in aortas from young and old *Lmna*^+/+^, *Lmna*^G609G/+^, and *Zmpste24*^−/−^ mice. Actin was measured as a loading control. The ages and mouse IDs are shown above each sample. **B** Bar graph shows the levels of total AKT (relative to actin) in young and old *Lmna*^+/+^, *Lmna*^G609G/+^, and *Zmpste24*^−/−^ mice. Mean ± SEM (*n* = 4 mice/group). ANOVA. ns, not significant. **C** Bar graph shows the levels of p-AKT-S473 (relative to actin) in young and old *Lmna*^+/+^, *Lmna*^G609G/+^, and *Zmpste24*^−/−^ mice. Mean ± SEM (*n* = 4 mice/group). ANOVA. *P < 0.05. **P < 0.01. **D** Bar graph shows the levels of p-AKT-S473 (relative to total AKT) in young and old *Lmna*^+/+^, *Lmna*^G609G/+^, and *Zmpste24*^−/−^ mice. Mean ± SEM (*n* = 4 mice/group). ANOVA. *P < 0.05. **P < 0.01.

To determine if AKT activity is reduced in the thoracic aorta of *Lmna*^G609G/+^ mice, we compared the phosphorylation of AKT at serine-473 [43] in separate cohorts of young and old wild-type (*Lmna*^+/+^), *Lmna*^G609G/+^, and *Zmpste24*^−/−^ mice. Relative to total amounts of AKT, AKT phosphorylation at serine-473 was lower in *Lmna*^G609G/+^ mice than in age-matched *Lmna*^+/+^ and *Zmpste24*^−/−^ mice (Fig. 7). Consistent with the reduced AKT activity in progerin-expressing cells, AKT phosphorylation was far lower in 14-week-old *Zmpste24*^−/−^*Lmna*^G609G/+^ mice than in age-matched *Zmpste24*^−/−^ mice (reduced by > 90%; ANOVA, *P* < 0.01).

## Discussion

The fact that progerin, an internally truncated and farnesylated prelamin A, triggers SMC loss in large arteries has been well documented [14, 16, 17]. A causal relationship between progerin and SMC loss is supported by the observation that the extent of SMC loss correlates with amounts of progerin expression [15, 25] and by the fact that progerin-mediated loss of SMCs can be reversed by extinguishing progerin synthesis [44]. However, it has never been clear why the loss of SMCs is pronounced in mice that express progerin (i.e., *Lmna*^G609G^ mice) but is absent in mice that express full-length farnesyl-prelamin A (i.*e*., *Zmpste24*^−/−^ mice, *Lmna*^L648R/L648R^ mice) [15, 38]. To understand this discrepancy, we compared the effects of progerin and full-length farnesyl-prelamin A expression in both cultured SMCs and genetically modified mouse models. We found that progerin and farnesyl-prelamin A, when expressed in identical amounts in cultured SMCs, result in identical levels of toxicity, as judged by the morphology of nuclear lamin meshwork and by the frequencies of misshapen cell nuclei, nuclear membrane ruptures, and cell death during mechanical stretching. In contrast to these cell culture observations, the effects of progerin and farnesyl-prelamin A in mouse models are distinct. In young mice, the levels of progerin in the thoracic aortas of *Lmna*^G609G/+^ mice and the levels of farnesyl-prelamin A in the thoracic aortas of *Zmpste24*^−/−^ mice are similar. In older mice, however, aortic levels of progerin in *Lmna*^G609G/+^ mice are greater than farnesyl-prelamin A levels in *Zmpste24*^−/−^ mice. That difference reflects an age-related accumulation of progerin in *Lmna*^G609G^ mice. In *Zmpste24*^−/−^ mouse aortas, farnesyl-prelamin A levels remain the same or decline with age. The accumulation of progerin in *Lmna*^G609G/+^ aortas could not be explained by changes in *Lmna* transcript levels (which remained stable or declined with age), implying that the accumulation of progerin in the thoracic aorta results from reduced turnover. Consistent with that idea, we found that the aortic levels of AKT activity, which phosphorylates A-type nuclear lamins at serine-404 [45] and targets prelamin A for degradation [42], is lower in *Lmna*^G609G/+^ mice than in wild-type and *Zmpste24*^−/−^ mice.

In an earlier study, we observed increasing levels of progerin in thoracic aortas of *Lmna*^G609G/+^ mice, raising the possibility of reduced levels of progerin turnover [21]. In the current study, we found that progerin accumulation in the aorta is accompanied by an accumulation of the other A-type nuclear lamins (lamin A, lamin C). In contrast, the levels of farnesyl-prelamin A and lamin C in the aorta of *Zmpste24*^−/−^ mice remain the same or fall with age. Progerin itself is responsible for the accumulation of A-type lamins. In progerin-expressing *Zmpste24*-deficient mice (i.e*., Zmpste24*^−/−^*Lmna*^G609G/+^ mice), we found a very substantial accumulation of farnesyl-prelamin A and lamin C—in addition to the accumulation of progerin.

Progerin also accumulates in the heart in *Lmna*^G609G^ mice, but in contrast to the findings in the aorta, the higher levels of progerin in the heart were not accompanied by widespread loss of cardiomyocytes. The crucial difference between the heart and the aorta is that the levels of *Lmna* expression are far lower in the heart. Thus, while progerin levels do accumulate in the heart of *Lmna*^G609G/+^ mice, the absolute levels of progerin in the heart never approached the extremely high levels observed in the aorta. In *Lmna*^G609G/+^ mice, the progerin:lamin B1 ratio is ~10-fold higher in the thoracic aorta than in the heart [15].

In a recent report [46], Hasper and colleagues examined “lifetimes” of nuclear lamins in a knock-in mouse model of HGPS. They found that the turnover of progerin in the aorta was reduced in the HGPS mice, consistent with our earlier observations [21]. However, it was not clear if there was an accumulation of progerin. In those studies, the abundance of the A-type nuclear lamins (lamin A, lamin C, progerin) was measured as a group in young (*n* = 3) and old (*n* = 2) HGPS mice. While the aortic levels of A-type lamins trended higher in HGPS mice than in wild-type mice, this difference was judged to be non-significant “due to variability across samples.” Higher levels of the A-type nuclear lamins were observed in the heart of HGPS mice, but since the A-type nuclear lamins were measured as a group it was not possible to determine whether the higher levels reflected an accumulation of progerin alone, an accumulation of lamin A and lamin C, or an accumulation of all three A-type nuclear lamins.

When we embarked on the current series of experiments, we initially suspected that the profound vascular disease in *Lmna*^G609G^ mice and its absence in *Zmpste24*^−/−^ mice could be due to different levels of lamin B1 in the aorta. In earlier studies [15, 21], we had observed that baseline levels of lamin B1 expression in the mouse thoracic aorta are very low and that the levels of lamin B1 in thoracic aortas of *Lmna*^G609G^ mice decreased as they aged. We found the very low levels of lamin B1 in aortas of *Lmna*^G609G^ mice to be intriguing because our cell culture studies had demonstrated that increasing the levels of lamin B1 expression reduced the toxic effects of progerin (e.g., abnormal meshwork, nuclear membrane ruptures, stress-induced cell death), whereas reducing levels of lamin B1 expression had the opposite effect [15, 21, 33]. In light of those observations, we initially suspected that we would find higher levels of lamin B1 in *Zmpste24*^−/−^ aortas than in *Lmna*^G609G^ mice, but this was not the case. The aortic levels of lamin B1 were low in both *Zmpste24*^−/−^ and *Lmna*^G609G^ mice, and they declined with age to a similar degree in both mouse models. Thus, the greater susceptibility of *Lmna*^G609G^ mice to vascular pathology could not be explained by lower levels of lamin B1 expression. We went on to define a straightforward explanation for the differences in vascular pathology: progerin levels in *Lmna*^G609G^ thoracic aortas accumulate with age, whereas farnesyl-prelamin A levels in *Zmpste24*^−/−^ thoracic aortas do not change or decline with age.

The different vascular phenotypes of *Zmpste24*^−/−^ and *Lmna*^G609G^ mice begged the question of whether very high levels of farnesyl prelamin A expression would be capable of triggering vascular disease. To address this question, we bred SMC-specific *Zmpste24* knockout mice harboring two *Lmna*^PLAO^ alleles (*Sm22α*-*CreZmpste24*^fl/fl^*Lmna*^PLAO/PLAO^). At 8 and 12 weeks of age, the aortic levels of farnesyl-prelamin A in *Sm22α*-*CreZmpste24*^fl/fl^*Lmna*^PLAO/PLAO^ mice were ~45% higher than in *Zmpste24*^−/−^ mice and by 18 weeks we found that that these mice had a substantial loss of aortic SMCs. Thus, supraphysiologic levels of farnesyl-prelamin A expression (mimicking the high levels of progerin in *Lmna*^G609G^ mice) result in the loss of aortic SMCs in vivo. Interestingly, the loss of SMCs was accompanied at late time points by reduced aortic levels of farnesyl-prelamin A and higher levels of mature lamin A. At first glance, that observation might seem paradoxical, but it was simply the consequence of variegated expression of the *Sm22α*-*Cre* transgene in aortic SMCs (which resulted in incomplete inactivation of the floxed *Zmpste24* allele). The incomplete inactivation of *Zmpste24* meant that aortas of *Sm22α*-*CreZmpste24*^fl/fl^*Lmna*^PLAO/PLAO^ mice contained a mixture of ZMPSTE24-deficient SMCs producing farnesyl-prelamin A and ZMPSTE24-positive cells producing mature lamin A. In those mice, the very high levels of farnesyl-prelamin A in the ZMPSTE24-deficient SMCs likely resulted in SMC death and declining levels of farnesyl-prelamin A, leaving behind healthy ZMPSTE24-expressing SMCs producing mature lamin A.

The finding that progerin levels increased progressively in *Lmna*^G609G^ aortas in the absence of increased levels of progerin transcripts implied that the turnover of progerin in the aorta of those mice was reduced. The phosphorylation of serine-404 in prelamin A is known to promote its degradation in lysosomes [42, 45]. Serine-404 is also phosphorylated in progerin [47]. Because AKT activity has already been shown to be perturbed in cell culture and in the heart of mouse models of HGPS [43, 48], we hypothesized that the progressive accumulation of progerin in *Lmna*^G609G^ aortas could be due to reduced phosphorylation by AKT. In cultured cells, we found that serine-404 was strongly phosphorylated in lamin A and farnesyl-prelamin A but weakly phosphorylated in progerin. The reduced phosphorylation of progerin could theoretically be due to differences in the conformation of progerin; however, the fact that progerin reduced the phosphorylation of farnesyl-prelamin A in *Zmpste24*^−/−^ SMCs suggested that progerin expression was accompanied by reduced AKT activity. We had initially hoped to quantify the phosphorylation of progerin in the aortas of mice, but antibodies against *mouse* lamin A/C phosphoserine-404 are not available. We did, however, examine AKT activity, as judged by phosphorylation of AKT at serine-473 [43, 48] in mouse aortas. AKT activity was significantly lower in thoracic aortas of *Lmna*^G609G/+^ mice than in thoracic aortas of wild-type or *Zmpste24*^−/−^ mice. Consistent with the ability of progerin expression to reduce AKT activity, AKT phosphorylation was lower in aortas of young and old *Zmpste24*^−/−^*Lmna*^G609G/+^ mice than in aortas of age-matched *Zmpste24*^−/−^ mice.

**Fig. 8:**
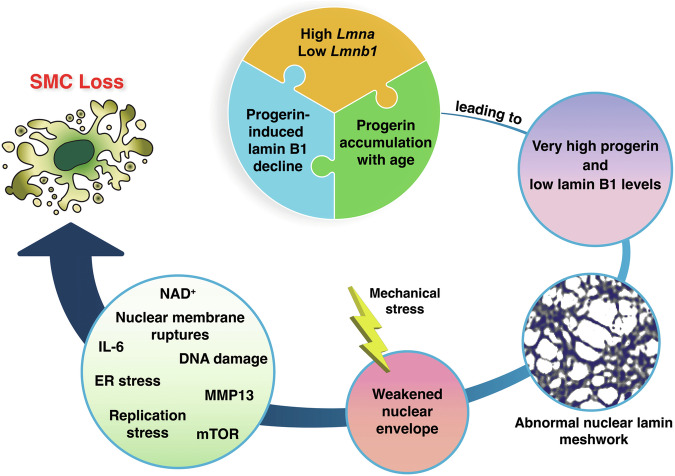
SMC loss in a mouse model of HGPS. A high *Lmna* and low *Lmnb1* expression profile in the mouse aorta results in a high lamin A:lamin B1 protein profile—ranking at the top compared to 10 other mouse tissues (e.g*.*,~10-fold higher than in the kidney) [15]. In the setting of HGPS, the high progerin:lamin B1 ratio increases with age and reaches extremely high levels (*e.g*.,~30-fold higher than in the kidney) [15]. This increase is due to two factors. First, progerin, the toxic molecule in HGPS, increases the turnover of lamin B1, thereby reducing lamin B1 levels in the aorta [21]. Lamin B1 acts in vitro to reduce the toxicity of progerin [21, 33]. Second, progerin causes the accumulation of progerin itself as well as the other A-type nuclear lamins (shown in the current studies). We propose that the very high progerin:lamin B1 profile in aortic SMCs underlies the morphologically abnormal nuclear lamin meshwork and the reduced integrity of the nuclear envelope [33]. In the setting of HGPS and mechanical stress (e.g., pulsatile flow in the aorta) [27], the structural abnormalities in the nuclear lamin meshwork lead to a constellation of molecular and cellular changes (e.g., nuclear membrane ruptures, ER stress, NAD^+^, IL-6, MMP13, mTOR, replication stress) that ultimately cause the loss of SMCs in the large arteries [15, 20–26].

In Fig. 8, we propose a model, based on our current findings, for vascular pathology in HGPS. Compared to other tissues, *Lmna* expression levels are high and *Lmnb1* levels are low in the thoracic aorta [15]. In the context of HGPS, this gene-expression pattern results in high levels of progerin and low levels of lamin B1 protein [15]. Progerin toxicity is known to be ameliorated by high levels of lamin B1 and is increased with low levels of lamin B1. As *Lmna*^G609G^ mice age, the high progerin–low lamin B1 protein profile becomes more pronounced [21], a consequence of both progerin accumulation and falling levels of lamin B1 expression. This results in gaps and irregularities in the nuclear lamina meshwork and reduced integrity of the nuclear membrane. Mechanical stress causes a breakdown of the weakened nuclear envelope (as judged by nuclear membrane ruptures in aortic SMCs), resulting in SMC DNA damage and pathogenic changes in organelle function and metabolic activity (e.g., ER stress, mTOR, NAD^+^) culminating in the loss of aortic SMCs [15, 20–26].

## Methods

### Mice

*Zmpste24*^−/−^ [49], *Lmna*^PLAO^ [40], *Lmna*^G609G^ [17], and *Zmpste24*^fl/fl^ [39] mouse strains have been described previously. The *Lmna*^PLAO^ directs all *Lmna* output into prelamin A synthesis. Thus, homozygous mice (*Lmna*^PLAO/PLAO^) produce only prelamin A (which is processed to mature lamin A) but no lamin C. The *Sm22α-Cre* (stock no. 017491) and ROSA^nT-nG^ (stock no. 023035) mouse strains were purchased from The Jackson Laboratory (Bar Harbor, ME). *Zmpste24*^−/−^*Lmna*^G609G/+^ (and littermate *Zmpste24*^−/−^ mice) were produced by intercrossing *Zmpste24*^+/–^*Lmna*^G609G/+^ mice. All mice were housed in a specific pathogen–free barrier facility with a 12-h light/dark cycle. The mice were provided pelleted mouse chow (NIH31) and water *ad libitum*. Both male and female mice were used for experimental studies. For all time course experiments, separate cohorts of mice were used at the different time points. Group sizes were based on published studies performing similar types of experiments. No animals were excluded for study unless the animals were in poor health or met pre-established criteria for premature euthanasia. Animals were randomly assigned to experimental groups (young and old). The ages of mice are reported in the figures.

### Immunohistochemical analysis of aortic tissue

Mice were perfused in situ with PBS followed by fixative solution (3% paraformaldehyde in PBS). The thoracic aorta was dissected free and incubated in fixative solution at 4 °C for 1–2 h. Aortic rings (~2-mm) from the proximal ascending aorta, proximal descending, and mid-descending aorta were embedded in OCT, and frozen sections (4–6-µm) collected onto glass slides. Tissue sections were incubated as described previously [15, 21] with antibodies and concentrations listed in Table S1. Nuclei were stained with Dapi. The stained sections were coded, and images were captured on a Zeiss LSM800 confocal microscope with a Plan-Apochromat 20×/0.8 NA objective. The coded images (tiff format) were imported into ImageJ, and the numbers of nuclei in the media were counted by trained observers blinded to genotype and expressed relative to media area.

### *Zmpste24*-deficient SMCs

Guide RNAs targeting exon 6 of *Zmpste24* (5′-GGTAAGGCTACCTGGAGGTG-3′) and (5′-ACAACATACACCTTAGTCAA-3′) were designed with Synthego’s gRNA design tool. Double-stranded gRNAs were subcloned into pX458-GFP CRISPR/Cas9 vector linearized with *Bbs*I. A Nucleofector II apparatus (Lonza) and the Cell line T Nucleofector kit (Lonza) were used to electroporate 2 µg of pX458 vectors containing the gRNAs into 2 × 10^6^ SMCs. After 48 h, transfected SMCs were cell sorted for the top 10% of GFP intensity by flow cytometry. Individual clones [20–30] were isolated by limiting dilution. Genomic DNA was extracted from SMC clones with the DNeasy kit (Qiagen) and subjected to PCR analysis. PCR primers flanking the gRNA cut sites (5′-ATTGCCTGTGTCTGCCCTTCTGCT-3′ and 5′-GAACACTGGTTTTGTTTTGCAGCC-3′) were used to amplify the gene fragment from the genomic DNA. Sequencing primer (5′-TCCACCAGCATGAACAAGGGTGTGT-3′) was used to verify the sequence deletion between the two guide RNA cut sites. Clonal cell lines were further tested by qPCR and western blotting to confirm the absence of ZMPSTE24 activity.

### *Lmna*-deficient SMCs

*Lmna-*deficient SMCs were generated in a similar fashion as *Zmpste24*-deficient SMCs. Guide RNAs targeting the 5′ and 3′ UTRs of *Lmna* (5′-GGATTGGCCGCTTCTGTGCG-3′) and (5′-CCAATCGCCGCACCTCTAGA-3′) were designed. Individual clones were isolated, and genomic DNA was extracted for sequencing. Deletion of the entire *Lmna* gene was confirmed by sequencing, qPCR, immunofluorescence, and western blotting.

### Measurement of nuclear membrane (NM) ruptures in live SMCs

SMCs stably expressing nls-GFP were seeded into 2-well chamber slides with #1.5 glass coverslip bottom (ThermoFisher Scientific) and cultured in complete media. To induce cell-cycle arrest, mitomycin C (1 µg/ml) was added to the media. The inhibition of mitosis greatly facilitated quantification of NM ruptures. Doxycycline was added to induce nuclear lamin expression for 24 h before examining the cells by confocal microscopy. The chamber slides were placed on a Zeiss LSM800 confocal laser-scanning microscope with a CO₂ and temperature-controlled stage, operated with Zen Blue 2.3 software (Zeiss). Cells were imaged for 48 h at 37 °C with 5% CO₂ using a Plan-Apochromat 20×/0.8 NA objective. Images from randomly selected fields were captured every 1–2 min. The ratio of cells with a NM rupture, identified by the presence of GFP in the cytoplasm, was calculated by dividing the number of cells with a rupture by the total number of cells in the field.

### High-resolution confocal fluorescence microscopy

A total of 50,000 cells were seeded in a chambered slide with a #1.5H (170 µm ± 5 µm) glass bottom (ibidi USA). After 48 h, the cells, with or without doxycycline treatment, were fixed using 4% paraformaldehyde in PBS for 10 min at room temperature, followed by permeabilization with 0.3% Triton X-100 in PBS for 10 min. Immunofluorescence microscopy was performed as previously described [33]. The antibodies and their concentrations are detailed in Table S1. Airyscan images were captured using a Zeiss LSM980 equipped with Airyscan2 in super-resolution (SR) imaging mode, with a scan speed of 5 and with a Plan-Apochromat 63×/1.4 NA oil-immersion objective. The excitation wavelengths and filter settings for each dye were chosen based on the integrated dye presets in ZEN Blue 2.3 software, and consistent settings were maintained throughout the imaging session. *Z*-stacks were acquired at an optimal section thickness of 0.14 µm, starting from near the glass bottom to the top of the nucleus. Images collected in SR mode were further enhanced with Airyscan Joint Deconvolution (Zeiss) with the following parameters: sample structure set to standard, maximum iterations at 10, and a quality threshold of 0.00. SR confocal fluorescence images were processed with ZEN Blue 2.3 software to create maximum intensity projection images from the equatorial plane to the top of the nucleus (furthest from the glass bottom).

### Quantification of lamina meshwork gaps

*Z*-axis images from the equator to the top of a nucleus were compiled and converted to 8-bit grayscale in ImageJ (NIH). Minor adjustments to brightness and contrast were made to optimize visualization of the nuclear lamina meshwork. The meshwork was outlined with the “Overlay” tool with a paintbrush set to 1-pixel width. A threshold was applied to define the meshwork, and the “Analyze Particles” function was used to quantify the areas of the gaps within the meshwork. A global scale was set using the original image’s scale bar. Meshwork gaps adjacent to the image borders were excluded from the analysis. The distribution of lamina meshwork gap areas was plotted for each nucleus. Nuclei exhibiting gap sizes greater than five times the average gap area were classified as abnormal.

### Measurement of cell death in stretched SMCs

Wild-type SMCs (1 × 10^5^) expressing human prelamin A, SMCs expressing human progerin, or ZMPSTE24-deficient SMCs expressing human prelamin A were seeded onto polydimethylsiloxane (PDMS) membranes and cultured for 48 h. The membranes were clamped in a custom-built biaxial cell stretching device [15] and stretched 3-mm at 0.5 Hz for 24 h. Dead cells detach from the membrane as judged by trypan blue staining. To measure the amount of surviving cells, membranes were rinsed with PBS and digested with 0.1 N NaOH. Protein content was measured with the DC protein assay kit (Bio-Rad).

### Statistical analysis

Sample sizes for individual experiments were based on the variance in preliminary studies. All bar graphs represent the mean of at least three independent experiments. Red circles in bar graphs show the average values of each experiment or values for individual animals. Statistical analyses were performed with Microsoft Excel for Mac 2021 and GraphPad Prism software. Experimental groups were analyzed by unpaired 2-tailed Student’s *t* test, or one-way and two-way ANOVA with Tukey’s multiple comparisons test. Statistical differences were considered significant when the *P* value was <0.05.

### Study approval

All animal studies were approved by UCLA’s Animal Research Committee (ARC#2014-002). All methods were performed in accordance with the relevant guidelines and regulations.

## Supplementary information

Original Data

Supplemental Materials

## Data Availability

All data are available in the main text or the Supplementary Materials. All unedited western blots are reported in the Supplementary file.
