# Supplementary material for: The accumulation of progerin underlies the loss of aortic smooth muscle cells in Hutchinson-Gilford progeria syndrome
Source: Cell Death Dis. 2025 Jul 24;16(1):557. doi: 10.1038/s41419-025-07853-0 (PMC12290114; doi:10.1038/s41419-025-07853-0)
Supplement: Supplementary file 1 — Original Data [file 41419_2025_7853_MOESM1_ESM.pdf]

Full unedited gel for Figure 2A.

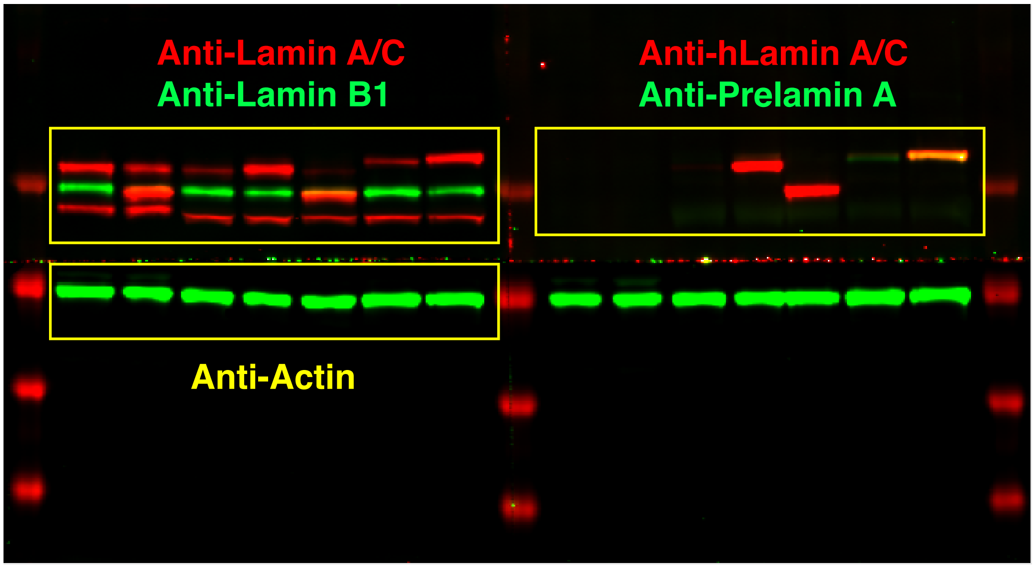

62 kDa
49 kDa
38 kDa
28kDa

Antibodies
Lamin A/C
Prelamin A (3C8)
hLamin A/C
Lamin B1
Tubulin

Full unedited gel for Figure 3A.

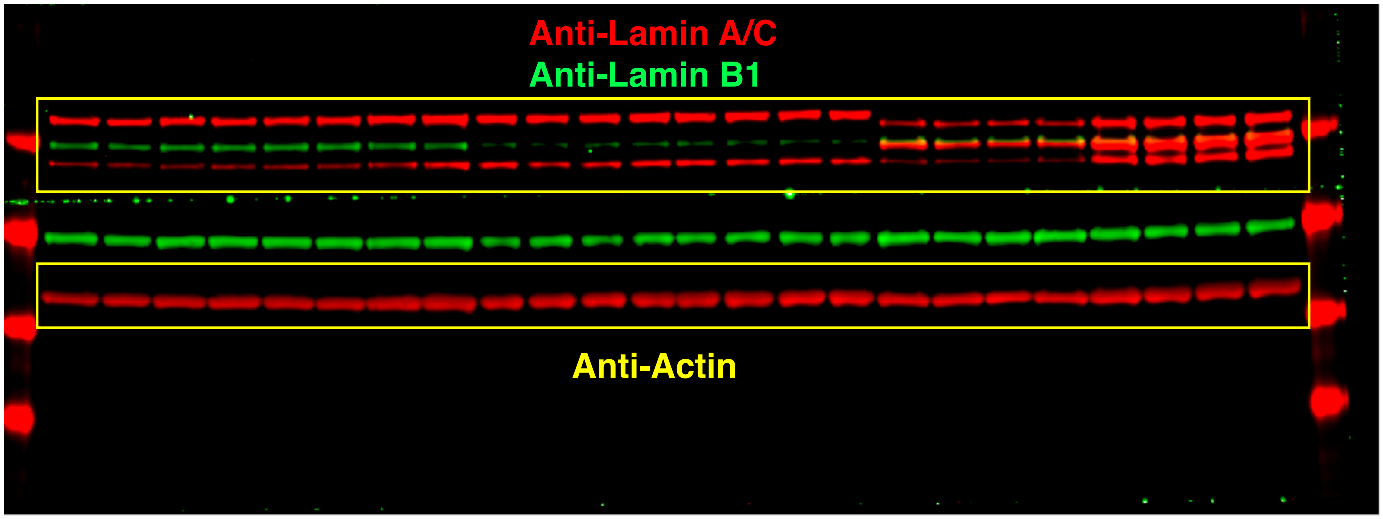

62 kDa
49 kDa
38 kDa
28kDa

Antibodies
Lamin A/C
Lamin B1
Actin
Tubulin

Full unedited gel for Figure 4A.

Merge

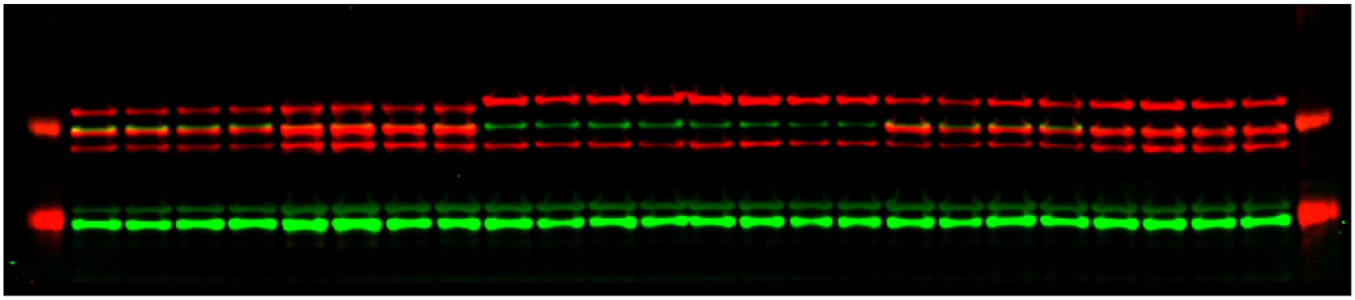

IR dye 800

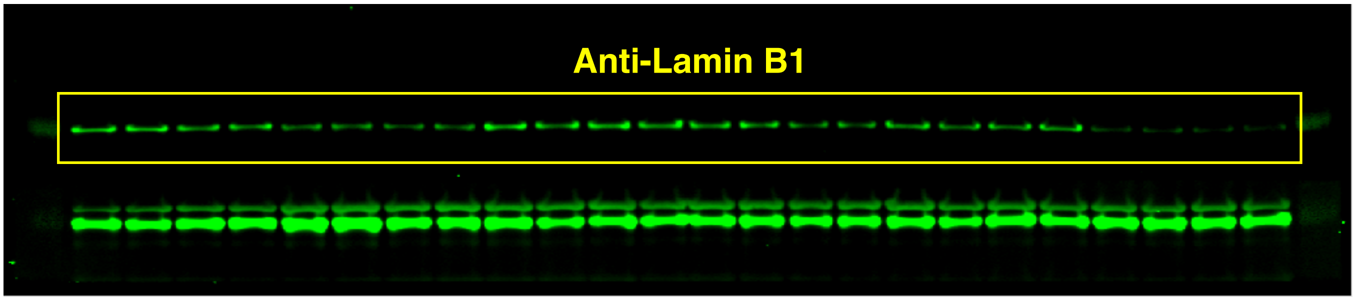

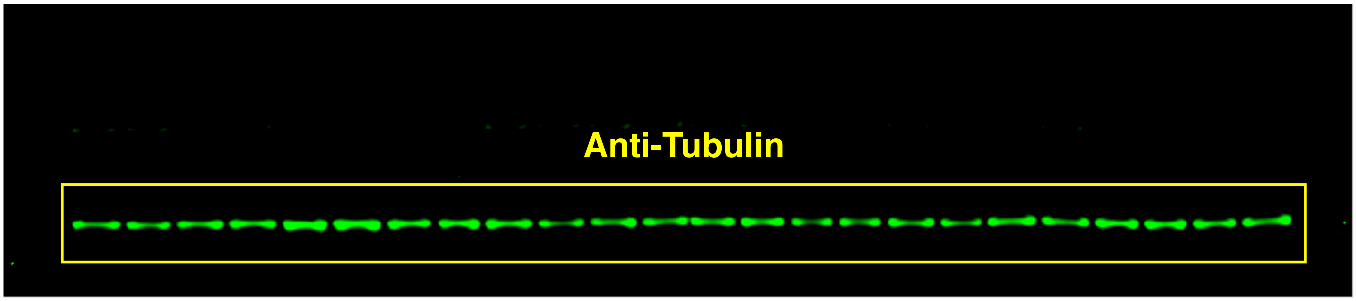

IR dye 680

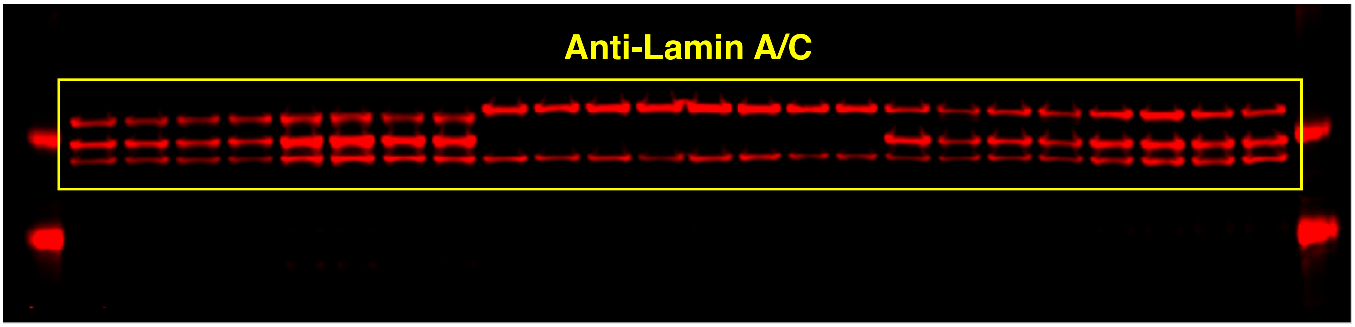

Antibodies
Lamin A/C
Prelamin A (3C8)
Tubulin

Full unedited gel for Figure 5A.

Merge

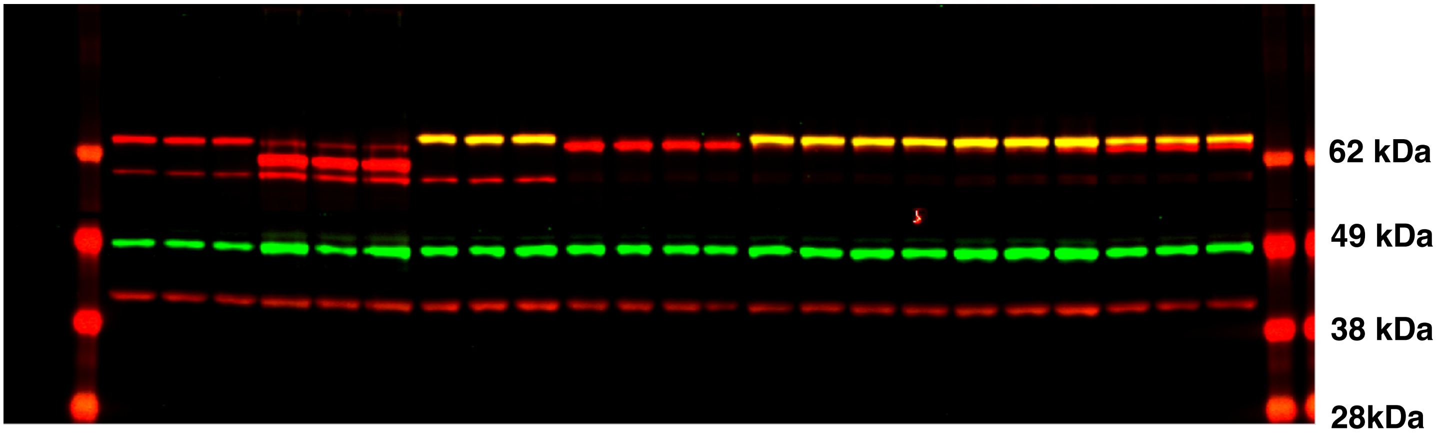

IR dye 800

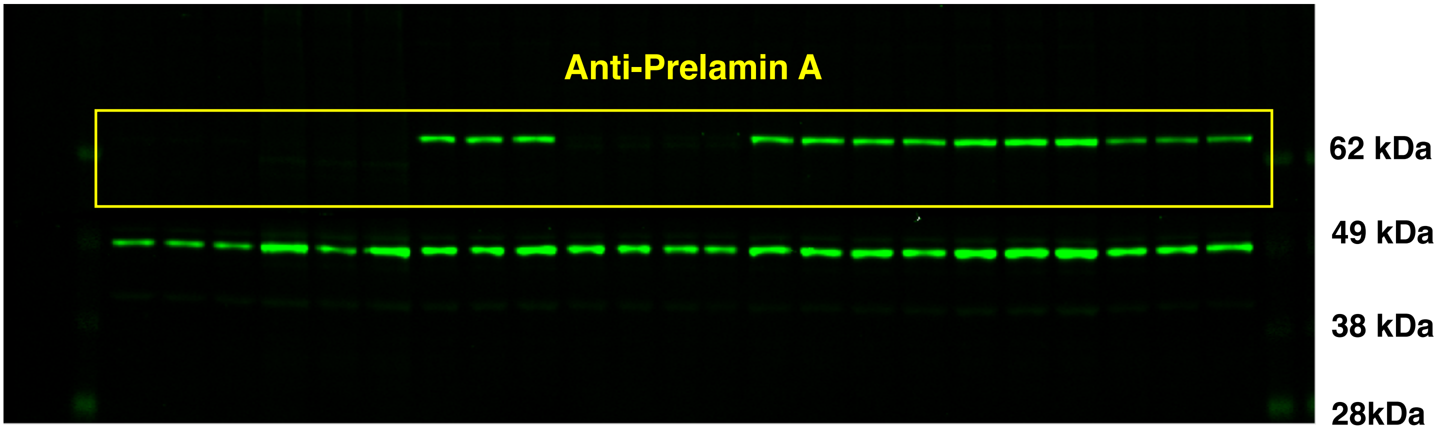

IR dye 680

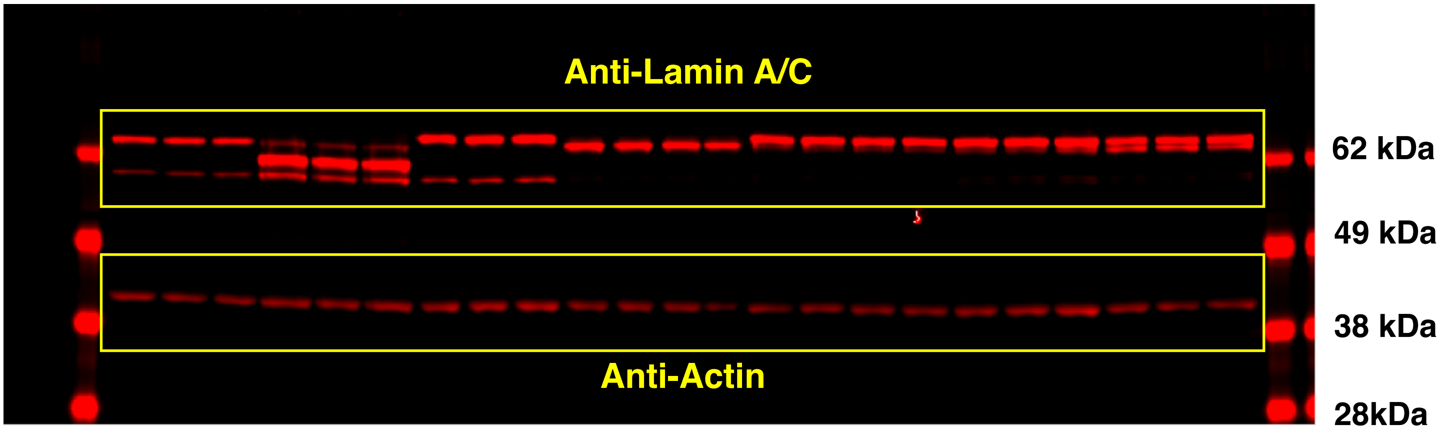

<u>Antibodies</u>
Lamin A/C
Prelamin A (3C8)
Tubulin
Actin

Full unedited gel for Figure 6A.

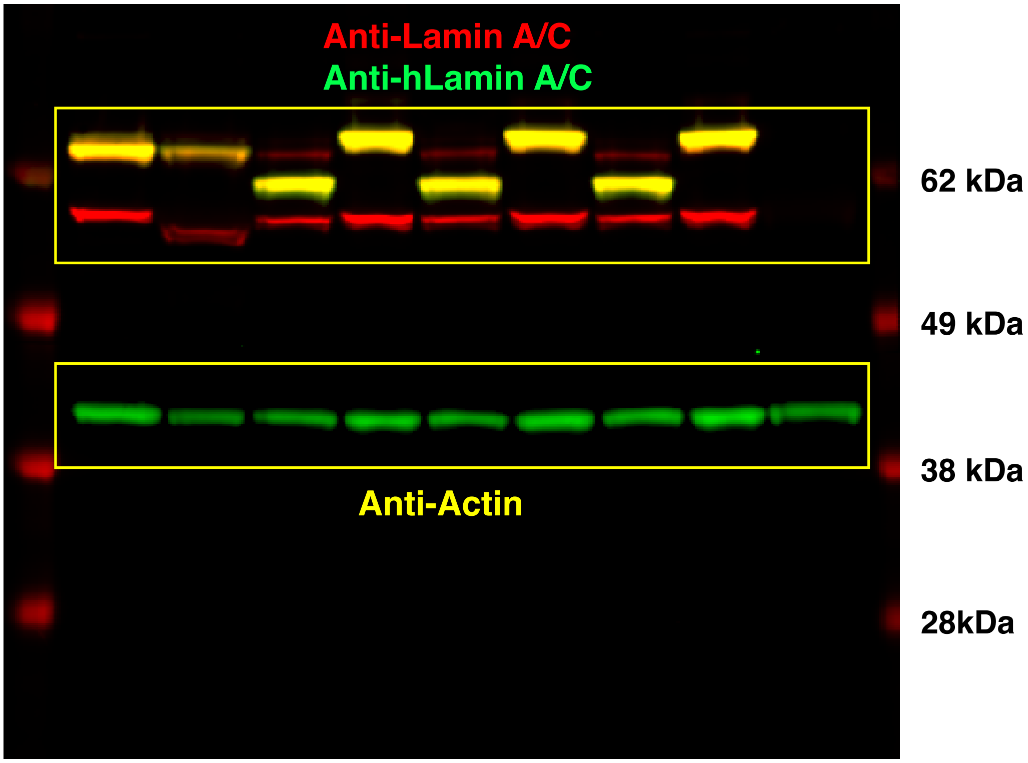

Antibodies
Lamin A/C
hLamin A/C
p-hLamin A/C (S404)
Actin

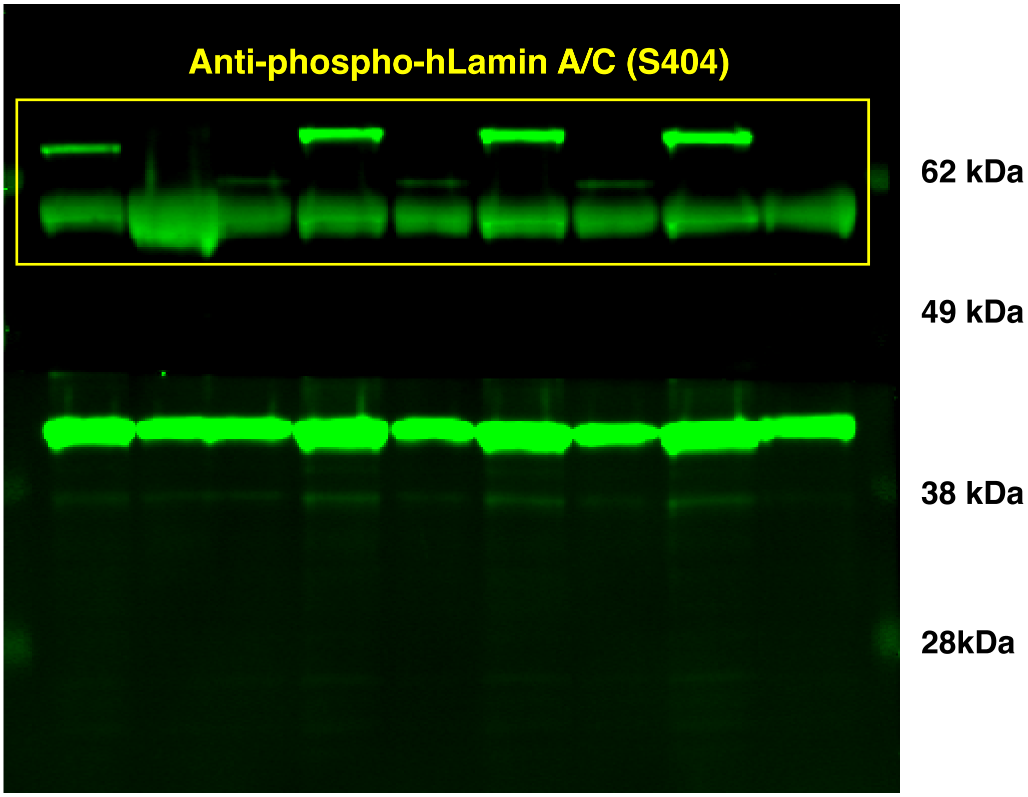

Full unedited gel for Figure 6C.

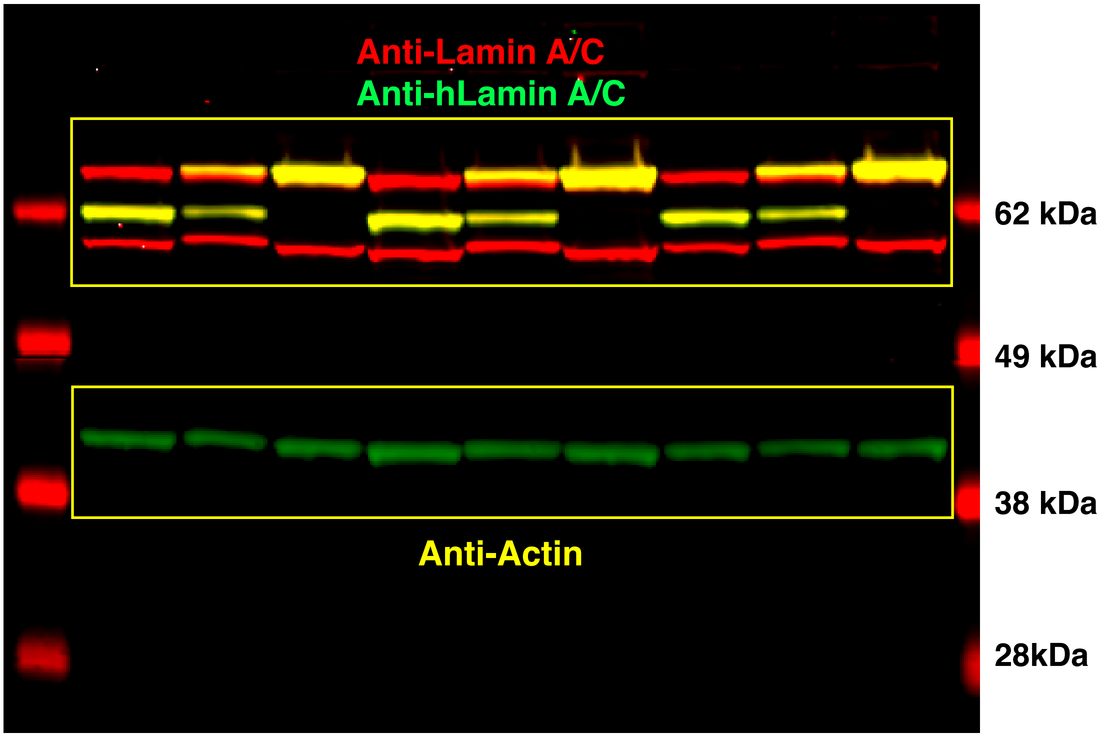

Antibodies
Lamin A/C
hLamin A/C
p-hLamin A/C (S404)
Actin

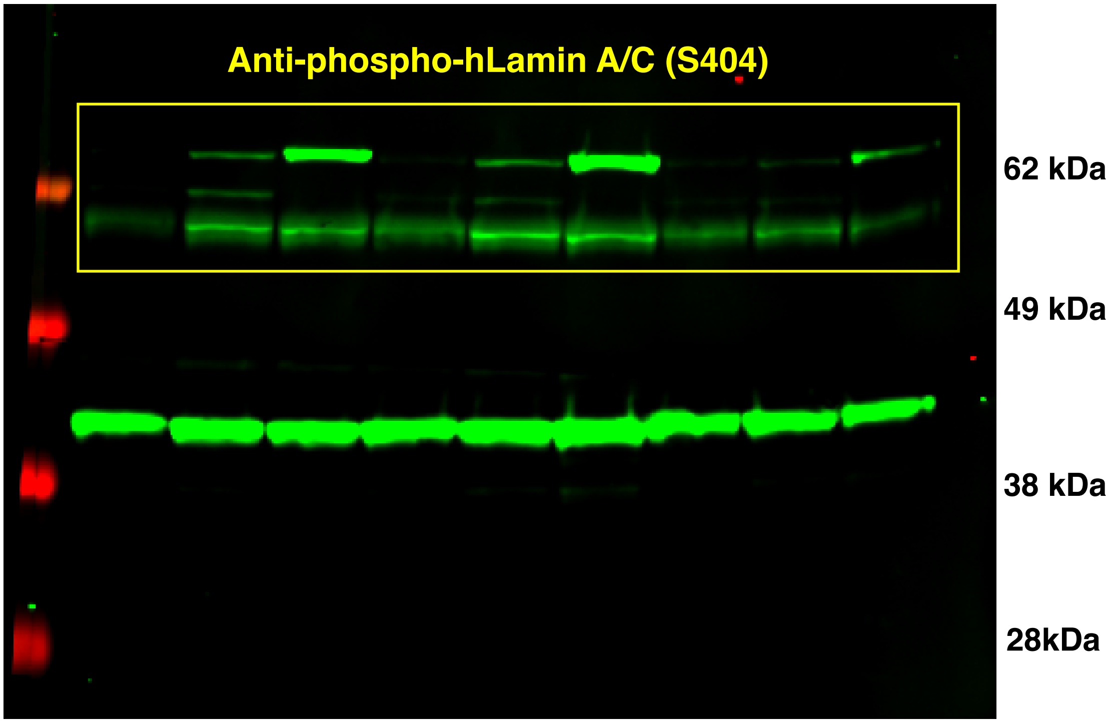

Full unedited gel for Figure 7A.

IR dye 680

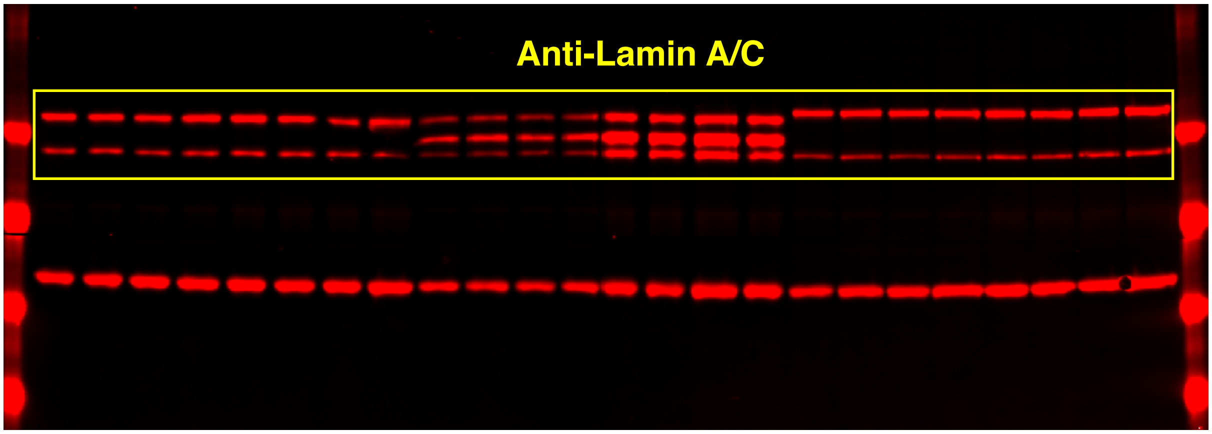

62 kDa
49 kDa
38 kDa
28kDa

IR dye 800

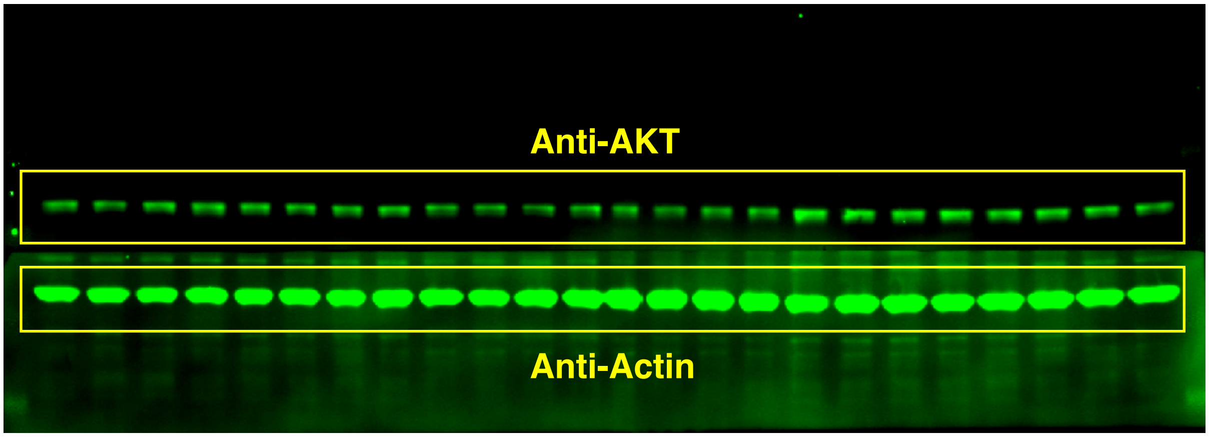

62 kDa
49 kDa
38 kDa
28kDa

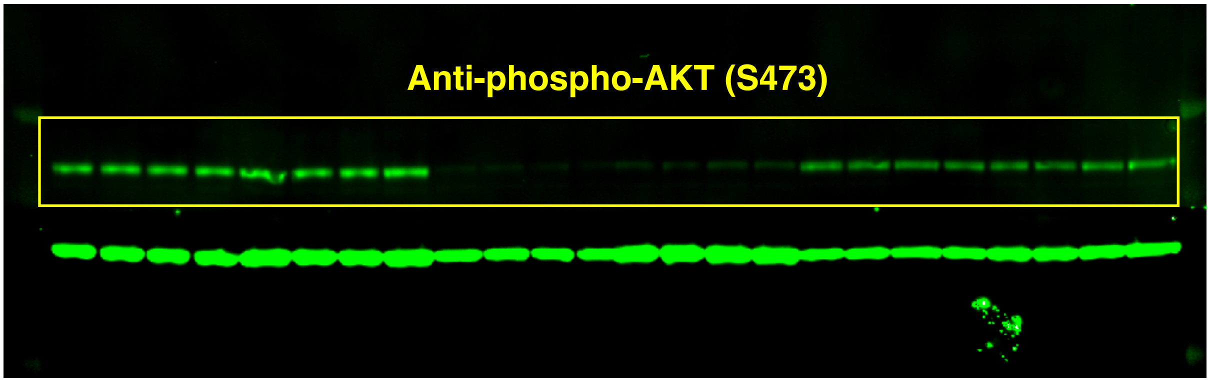

62 kDa
49 kDa
38 kDa
28kDa

Antibodies
Lamin A/C
AKT (pan)
p-AKT (S473)
Actin

Full unedited gel for Figure S4A.

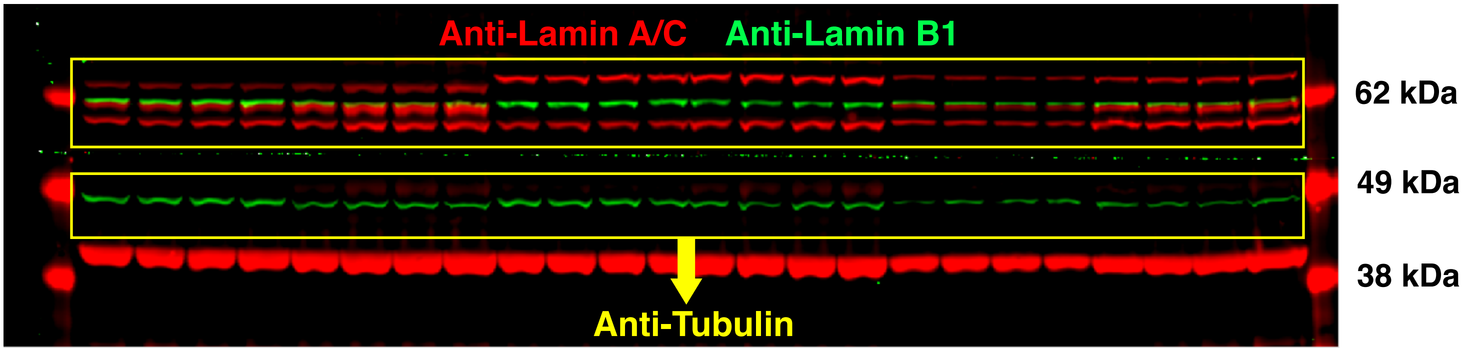

Antibodies
Lamin A/C
Lamin B1
Tubulin
Actin

Full unedited gel for Figure S5A.

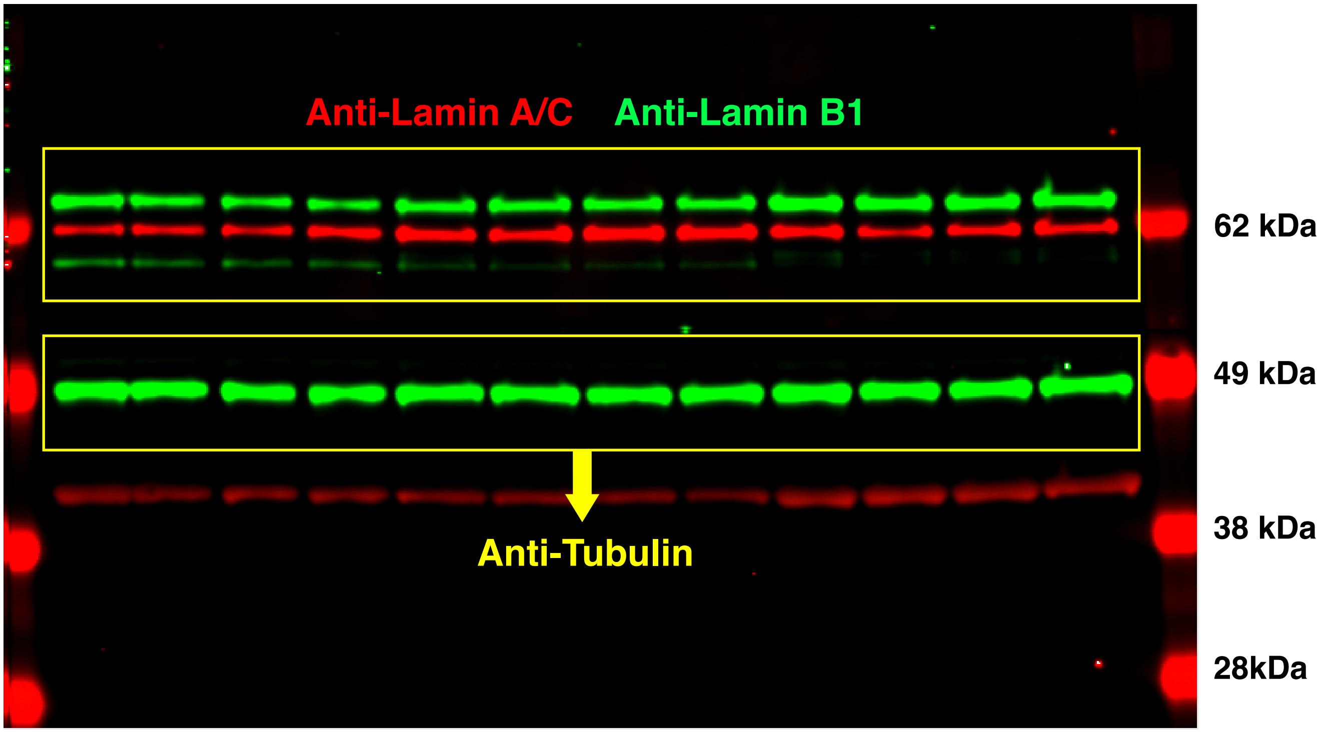

Antibodies
Lamin A/C
Lamin B1
Tubulin
Actin

Full unedited gel for Figure S5B.

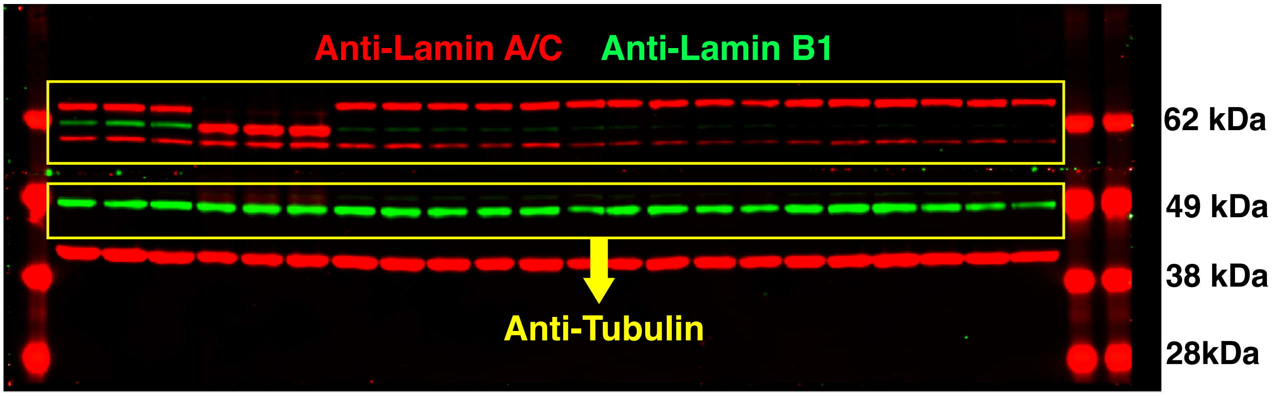

Antibodies
Lamin A/C
Lamin B1
Tubulin
Actin

Full unedited gel for Figure S5A.

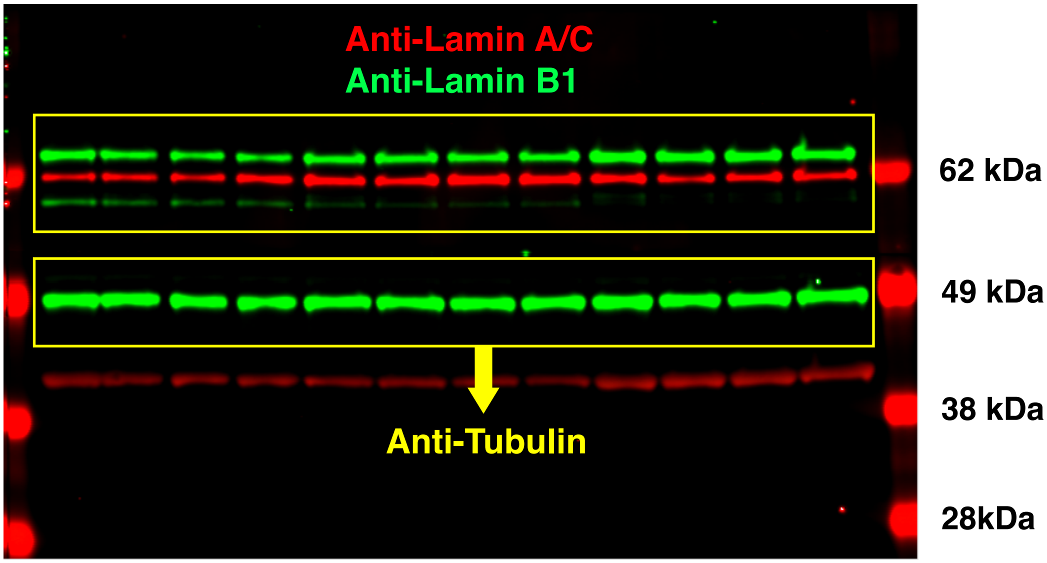

Antibodies
Lamin A/C
Lamin B1
Tubulin
Actin

Full unedited gel for Figure S5B.

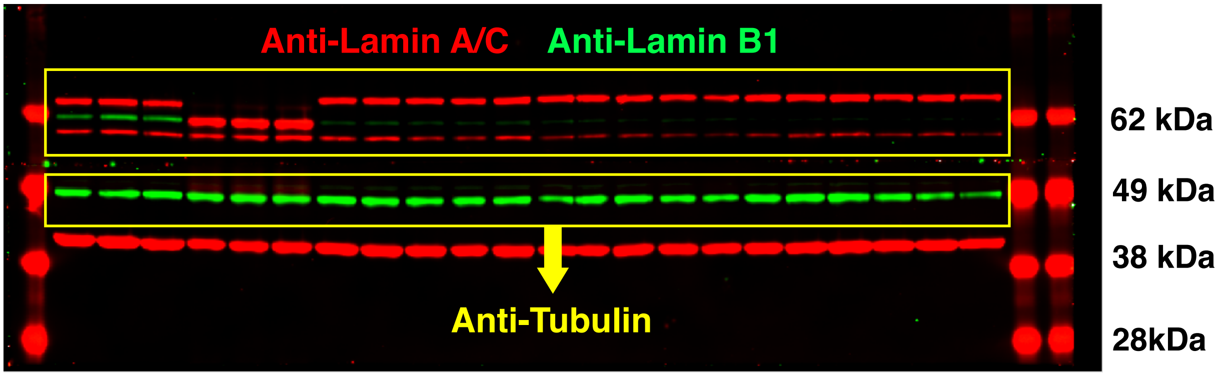

Antibodies
Lamin A/C
Lamin B1
Tubulin
Actin
